# Entropy‐Driven Physical Amplification in Multivalent Biosensing

**DOI:** 10.1002/advs.77266

**Published:** 2026-08-25

**Authors:** Yuhan Peng, Xiuyang Xia, Ran Ni

**Affiliations:** ^1^ School of Chemistry Chemical Engineering and Biotechnology Nanyang Technological University Singapore Singapore; ^2^ Department of Physics Southern University of Science and Technology Shenzhen Guangdong China; ^3^ Center for Complex Flows and Soft Matter Research Southern University of Science and Technology Shenzhen Guangdong China; ^4^ Arnold Sommerfeld Center for Theoretical Physics and Center for NanoScience Department of Physics Ludwig‐Maximilians‐Universität München München Germany

**Keywords:** multivalent linkers, superselectivity, ultrasensitive detection

## Abstract

Sensitive detection of low‐abundance molecular targets is widely assumed to require enzymatic amplification, such as PCR, to achieve low detection limits. In amplification‐free platforms, sensitivity is traditionally constrained by equilibrium binding affinity. Here, we show theoretically that multivalent linker entropy provides a distinct physical route to exponential sensitivity enhancement in purely equilibrium‐based sensing architectures. Using a statistical‐mechanical theory supported by grand canonical Monte Carlo simulations, we demonstrate that redistributing a fixed total interaction strength over increasing linker valency exponentially lowers the adsorption threshold. This scaling emerges not from stronger energetic affinity, but from the rapid growth of combinatorial binding configurations, identifying entropy as a physical mechanism that amplifies the adsorption response. Crucially, this threshold shift requires no increase in total binding strength, enabling ultrasensitive responses without enzymatic replication. This prediction is consistent with the ultrasensitive, amplification‐free detection demonstrated experimentally in DNA‐functionalized colloidal systems [*Proc. Natl. Acad. Sci*. USA 120, (2023): e2305995120], whose physical origin had remained unexplained. Our theory identifies entropy‐driven combinatorial effects as a plausible mechanism for this high sensitivity. More broadly, it establishes combinatorial linker entropy as a design principle for high‐sensitivity, amplification‐free detection.

## Graphical abstract

Detecting scarce molecules usually demands enzymatic amplification such as PCR. A statistical‐mechanical theory now reveals a purely physical alternative: distributing a fixed binding strength over more linker arms exponentially lowers the concentration at which multivalent sensors switch on. This gain stems from combinatorial entropy, not stronger bonds, pointing toward ultrasensitive, amplification‐free detection.

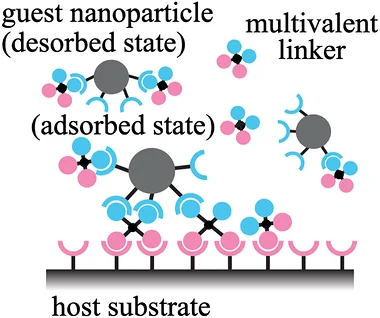

## 1 Introduction

Sensitive detection of biomarkers and pathogens requires achieving low detection limits at physiologically relevant analyte concentrations. In amplification‐based platforms such as PCR, extraordinary sensitivity is obtained through exponential molecular replication [1, 2]. In contrast, amplification‐free biosensing systems must rely directly on binding thermodynamics and collective molecular interactions to generate detectable signals [3, 4]. Understanding how such systems can approach amplification‐level sensitivity without enzymatic replication remains a fundamental physical challenge [4].

Multivalent interactions provide a general physical mechanism for generating nonlinear and highly cooperative responses in systems composed of many weak binding units [5, 6, 7]. By distributing interactions over multiple binding sites, multivalency allows individually weak bonds to collectively stabilize macroscopic states, leading to sharp transitions in adsorption, adhesion, and molecular recognition [8]. In the context of surface adsorption, statistical‐mechanical frameworks have shown that multivalent binding can produce superlinear, and even superselective, responses to changes in environmental parameters [7]. Importantly, existing studies have primarily focused on how multivalency sharpens response slopes, enabling discrimination based on receptor density or target abundance, whereas much less is known about how multivalency controls the location of response thresholds themselves, particularly under constraints on total interaction strength.

In many experimentally relevant systems, multivalent recognition does not occur through direct adsorption alone, but instead proceeds via linker‐mediated architectures, in which soluble molecules bridge ligands on one object to receptors on another. Such bridging motifs are ubiquitous across bioanalytical and soft‐matter platforms, including antibody‐based sandwich immunoassays [9, 10], DNA‐mediated nanoparticle assays [11, 12, 13] and whole‐genome detection [14, 15]. A compelling experimental demonstration of the sensitivity achievable in such architectures is provided in Ref. [14], showing that DNA‐functionalized colloidal particles can detect bacteria at extraordinarily low ssDNA concentrations without any enzymatic amplification. In that system, long single‐stranded DNAs act as multivalent linkers bridging colloidal particles to target sequences distributed along the bacterial genome, driving cooperative colloidal aggregation at vanishingly low target concentrations. Despite its remarkable experimental performance, the physical mechanism underlying this extraordinary sensitivity has remained unexplained. From a physical perspective, linker‐mediated interactions fundamentally alter the structure of multivalent binding by introducing an additional degree of freedom: linkers can form productive bridges connecting the two surfaces, or remain non‐productively sequestered by dangling from either side. This competition between bridging and sequestration gives rise to response behaviors that differ qualitatively from direct multivalent adsorption, including nonmonotonic responses characterized by a low‐concentration “turn‐on” threshold and a high‐concentration “turn‐off” threshold that together define a finite operating window [10, 16].

These features raise a fundamental question concerning multivalent interacting systems under constrained interaction strength. Specifically, when the *total* binding strength between two objects is fixed, what controls the position of the adsorption threshold? Is the threshold set primarily by energetic affinity, or can collective and entropic effects associated with multivalency qualitatively reshape it? In linker‐mediated architectures, this question becomes particularly acute because linker valency provides a natural means of redistributing a fixed interaction strength over multiple binding arms, necessarily weakening individual bonds while expanding the space of accessible binding configurations, thereby introducing an intrinsic trade‐off between energetic stabilization and combinatorial entropy.

Here we address this question by developing a minimal statistical‐mechanical framework for linker‐mediated multivalent adsorption. Combining a Langmuir‐like adsorption description [7, 15], a theory for multivalent bridging [17], and grand canonical Monte Carlo simulations, we systematically isolate the role of linker valency under the constraint of fixed total binding strength. We find that redistributing a fixed interaction strength over an increasing number of linker arms can exponentially shift the adsorption threshold to lower linker concentrations. This strong and counterintuitive scaling does not originate from enhanced energetic affinity, but instead from the rapid growth of the combinatorial entropy associated with multivalent binding configurations, revealing an entropy‐dominated mechanism for threshold position.

## 2 Results

### 2.1 Model and Simulations

**FIGURE 1:**
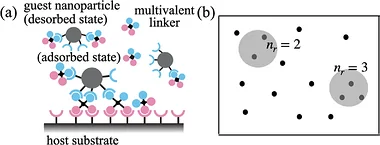
Multivalent linker‐mediated adsorption of guest particles. (a) Schematic illustration of the multivalent linker‐mediated interaction between guest nanoparticles and the host substrate. A guest particle is in the *adsorbed* state when at least one linker bridges it to the substrate, and in the *desorbed* state otherwise. (b) Schematic of the GCMC simulation setup. Black dots represent immobile receptors randomly distributed on the substrate. Large gray circles indicate guest particles, each of which can interact only with receptors located within its own shaded region via linkers.

We consider a coarse‐grained model where guest particles adsorb onto a host substrate via multivalent linkers whose two distinct ends bind to ligands on the guest particles and receptors on the host substrate, respectively (Figure 1a). Following a Langmuir‐like framework [7], we consider a substrate site with $n_{r}$ immobile receptors that can accommodate at most one guest particle. Thus, each site exists either in a *desorbed* state, where no linker bridge is formed, or in an *adsorbed* state, where at least one linker bridges between the guest particle and receptors on the site. A guest particle is coated with $n_{l}$ mobile ligands, where the mobility of ligands allows them to potentially bridge with any receptor at its corresponding site [15]. Linkers are treated as an ideal gas of density $\rho =e^{\beta \mu }/\Lambda^{3}$, with the chemical potential μ, de Broglie wavelength Λ, and the inverse thermal energy $\beta =1/k_{B}T$, where $k_{B}$ and T are the Boltzmann constant and system temperature, respectively. Each linker possesses $\kappa_{l}$ and $\kappa_{r}$ binding ends for ligands and receptors, with individual binding free energies $f_{l}$ and $f_{r}$, respectively. The formation of a bridging linker is penalized by a mean‐field configurational free energy cost $f_{\mathrm{cnf}}$.

We formulate the grand potential of the system in both adsorbed and desorbed states. Our approach is based on the framework in Ref. [17], which has been validated by thermodynamic integration for systems with explicit multivalent linkers. The central idea is to treat each linker as interacting only with receptors on a single site and ligands on the associated guest particle, assuming that the linker size is small compared to the site and guest particles. Thus, linkers mediate an effective interaction between the host and the guest, without coupling with other sites. In the adsorbed state, besides free linkers in solution, each linker may dangle from a receptor, dangle from a ligand, or bridge between receptors and ligands, thereby connecting the substrate and the particle. By applying the saddle‐point approximation, we determine the equilibrium number of linkers in each state: $M_{l}$ for those dangling on ligands, $M_{r}$ for those dangling on receptors, and Q for those that form bridges. The resulting grand potential for the adsorbed state is [see Supporting Information]

(1) $$\beta F_{\mathrm{ad}}=\sum_{i\in \{l,r\}}\left(n_{i}\log{\bar{n}}_{i}-{\bar{n}}_{i}-M_{i}\right)-Q,$$

where ${\bar{n}}_{i}$ denotes the number of unbound ligands (i=l) or receptors (i=r) in the adsorbed state. Similarly, for the desorbed state, only dangling linkers exist, as no bridges can form when the guest particle is absent. The grand‐canonical potential in the desorbed state is given by

(2) $$\beta F_{\mathrm{dp}}=\sum_{i\in \{l,r\}}\left(n_{i}\log{\bar{n}}_{i}^{\prime }-{\bar{n}}_{i}^{\prime }-M_{i}^{\prime }\right),$$

where $M_{l}^{\prime }$ and $M_{r}^{\prime }$ represent the numbers of linkers dangling from ligands and receptors, respectively, and ${\bar{n}}_{i}^{\prime }$ represents the number of unbound ligands or receptors when the guest particle is infinitely far from the site. M, Q, and $\bar{n}$ are numerically calculated by solving a set of self‐consistent equations, in which the convergence of the solution for arbitrary linker valency is ensured [17].

We assume that adsorption events on individual substrate sites are independent, with no interaction between particles adsorbed on adjacent sites. For each site, we take the desorbed state as the reference, and define the referenced partition function of the adsorbed state as $q=e^{-\beta (F_{\mathrm{ad}}-F_{\mathrm{dp}})}-1$ [18]. The averaged grand partition function of all sites is then given by $\Xi ={\left\langle 1+z_{g}q\right\rangle }_{\left\langle n_{r}\right\rangle }$, where $z_{g}$ is the activity of guest particles in the bulk solution, proportional to the particle density and adsorption volume [7, 15]. ${\left\langle \cdot \right\rangle }_{\left\langle n_{r}\right\rangle }$ calculates the average over the Poisson distribution of uncorrelated, immobile receptors, characterized by the mean $\left\langle n_{r}\right\rangle$. The probability of nanoparticle adsorption follows a Langmuir‐type isotherm:

(3) $$\theta :=\frac{\partial \log\Xi }{\partial \log z_{g}}={\langle \frac{z_{g}q}{1+z_{g}q}\rangle }_{\left\langle n_{r}\right\rangle }.$$

To validate our theoretical prediction and provide a parameter mapping for future studies, we performed grand canonical Monte Carlo (GCMC) simulations of guest nanoparticles in contact with the host substrate coated with explicit receptors. Due to the low density and the multivalent nature of guest particles in most experiments, the equilibration time for 3D simulations is extremely long. Therefore, we simulate a monolayer of guest particles on the substrate where the guest particles can be modeled as 2D hard disks, and they are in chemical equilibrium with a bulk 3D reservoir above. The adsorption and desorption of guest particles are treated as the insertion and deletion of particles from the reservoir in the simulation [see Supporting Information]. Guest particles are modeled as hard disks with diameter σ=1 on a two‐dimensional (2D) substrate in chemical equilibrium with a reservoir at chemical potential $\mu_{g}$ [15, 19] (see Figure 1b). The activity of guest particles is given by $z_{g}=e^{\beta \mu_{g}}\pi \sigma^{2}h_{0}/\left(4\Lambda^{3}\right)$, where $h_{0}=1$ is the thickness of the adsorption layer. Immobile receptors are randomly distributed on the substrate following a Poisson distribution with number density $\rho_{r}$. Mobile ligands on each guest particle can interact with receptors located within the area underneath the particle. Guest particles move on a 2D plane of box length L with periodic boundary conditions and interact via hard‐disk repulsion. The maximum number of adsorption sites is set to $N_{\max}=4L^{2}/\left(\pi \sigma^{2}\right)$, and the adsorption probability is calculated as $\theta =\left\langle N_{g}\right\rangle /N_{\max}$, where $\left\langle N_{g}\right\rangle$ is the average number of adsorbed guest particles.

We focus on how linker multivalency modulates two key features of the adsorption systems: sensitivity and selectivity. Here, sensitivity refers to the lowest detectable value of a given environmental parameter $\chi^{*}$ at which adsorption becomes significant. Operationally, we define the sensitivity threshold $\chi^{*}$ by the condition $\theta (\chi^{*})=0.1$. Selectivity quantifies the degree to which adsorption responds to changes in χ, and is characterized by the selectivity parameter $\alpha_{\chi }=d\log\theta /d\log\chi$. When $\alpha_{\chi }>1$, the adsorption increases superlinearly with respect to χ with $\theta ∼\chi^{\alpha_{\chi }}$. Conversely, $\alpha_{\chi }<-1$ indicates that the desorption also proceeds in a superlinear fashion. Both situations are referred to as χ‐dependent superselectivity.

### 2.2 Ultrasensitive ρ‐Response

To investigate how linker valency κ affects the adsorption of guest nanoparticles, we perform both theoretical analysis and GCMC simulations, considering symmetric linkers with $\kappa =\kappa_{l}=\kappa_{r}$. We fix the overall binding strength to isolate the effect of linker valency so that any change in the adsorption of nanoparticles arises solely from the combinatorial entropy associated with multivalency. Accordingly, in all calculations we fix the total binding strength as $f_{\mathrm{tot}}=\kappa f_{l}=\kappa f_{r}$.

**FIGURE 2:**
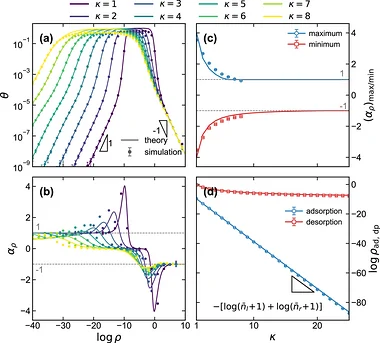
ρ‐dependent superselectivity and ultrasensitivity. Parameters: $\kappa \beta f_{l}=\kappa \beta f_{r}=-5$, $n_{l}=\left\langle n_{r}\right\rangle =10$, $\beta f_{\mathrm{cnf}}=2$, and $z_{g}=10^{-5}$. Symbols denote GCMC simulation results; solid curves are the theoretical predictions from Equation (3). (a) Adsorption probability θ versus logρ for linker valencies κ=1–8. Increasing κ at fixed total binding strength shifts the adsorption onset to exponentially lower linker densities. (b) Selectivity $\alpha_{\rho }=d\log\theta /d\log\rho$ versus logρ, showing superselective ($|\alpha_{\rho }|>1$) regimes on both the adsorption and desorption sides [see Supporting Information]. The symbols are computed from simulation data with the Savitzky–Golay filter method. (c) Peak selectivity ${\left(\alpha_{\rho }\right)}_{\max}$ and minimum selectivity ${\left(\alpha_{\rho }\right)}_{\min}$ versus κ, illustrating the decay of superselectivity with increasing valency. (d) The adsorption threshold decreases linearly in κ on a logarithmic scale, with the predicted slope given by Equation (5). The desorption threshold converges at large κ.

As shown in Figure 2a, the adsorption θ increases linearly with ρ at low concentrations [see Supporting Information], followed by a pronounced superlinear regime. Remarkably, at high linker concentrations, after a saturation plateau, θ begins to decrease as ρ increases further, which is a distinct feature of linker‐mediated interactions [20, 21]; the leading‐order scalings in these regimes are summarized in Table S1. The corresponding selectivity parameter $\alpha_{\rho }$, shown in Figure 2b, reveals that for small and moderate linker valency κ, both adsorption and desorption transitions are superselective, i.e., the maxima and minima of $\alpha_{\rho }$ exceed 1 in magnitude ($\alpha_{\rho ,\max}>1$, $\alpha_{\rho ,\min}<-1$). Interestingly, as κ increases, the selectivity weakens: $\alpha_{\rho ,\max}$ decreases and $\alpha_{\rho ,\min}$ increases, both approaching 1 and −1 in the large valency limit (Figure 2c). This is because, as the linker valency increases, fewer linker molecules are required to bridge, and eventually a single linker may suffice to fully bridge the host and guest, so that the superselectivity with respect to linker concentration vanishes. In the limit of zero particle activity $z_{g}\to 0$ and strong individual binding $f_{l/r}\to -\infty$, our mean‐field analysis predicts that the maximal selectivity decays as $\alpha_{\rho ,\max}\approx \max\left[\left(\min\left(n_{l},n_{r}\right)\right)/\kappa ,1\right]$ (Figure S1), indicating that the number of bridges ultimately limits the attainable superselectivity. When κ exceeds $\min(n_{l},n_{r})$, the system loses its superselective response to linker concentration. In realistic regimes of finite $f_{l/r}$ and $z_{g}$, the selectivity is reduced even further, consistent with GCMC simulations.

In addition to selectivity, linker valency also strongly influences the adsorption and desorption sensitivity thresholds, $\rho_{\mathrm{ad}}$ and $\rho_{\mathrm{dp}}$. As shown in Figure 2d, $\rho_{\mathrm{ad}}$ decreases exponentially with increasing κ, whereas $\rho_{\mathrm{dp}}$ decreases and then reaches a plateau at large κ. Since the overall binding strength is fixed, these trends arise from the growing combinatorial entropy introduced by linker multivalency. To elucidate the underlying physics, we examine the large‐κ limit at a single site.

At the adsorption sensitivity threshold $\rho_{\mathrm{ad}}$, both adsorbed and bridging linkers are extremely sparse at large κ (Figure S2), because only a very small number of linkers are sufficient to fully bind the guest particle to the site. Thus, the effective interaction between a guest particle and its site mainly depends on the number of unbound ligands and receptors [see Supporting Information]

(4) $$\beta F_{\mathrm{eff}}≃\sum_{i=l,r}\left[n_{i}\log\left(\frac{{\bar{n}}_{i}}{n_{i}}\right)-\left({\bar{n}}_{i}-n_{i}\right)\right].$$

Under the Poisson distribution of receptor numbers, Equation (3) and (4) yield the adsorption sensitivity threshold [see Supporting Information]

(5) $$\log\rho_{\mathrm{ad}}∼-\lim_{\kappa \to +\infty }\left[\log\left({\bar{n}}_{l}+1\right)+\log\left({\bar{n}}_{r}+1\right)\right]\kappa .$$

This shows that $\rho_{\mathrm{ad}}$ decreases exponentially with increasing linker valency κ, with the exponent given by the constant $-\left[\log\left({\bar{n}}_{l}+1\right)+\log\left({\bar{n}}_{r}+1\right)\right]$ at the large κ limit, which agrees quantitatively with GCMC simulations (Figure 2d; see also Figure S3). Thus, as the total binding strength $\kappa f_{l/r}$ is fixed, this exponential scaling reflects the dominant contribution of the combinatorial entropy associated with linker multivalency. Physically, this threshold reduction does not arise from any change in the bonds themselves. The total binding strength is held fixed, and forming one bond leaves the affinity of the others unchanged, so the mechanism is neither allosteric nor enzyme‐like [22, 23]. What grows with valency is instead the number of accessible binding configurations. Recruiting a linker from solution costs configurational entropy only once; once it is localized in the contact region, its remaining arms bind with no further configurational cost. This result shows that, even at fixed overall binding strength, increasing κ can exponentially lower the adsorption threshold $\rho_{\mathrm{ad}}$ (Figure S4), which underlies the ultrasensitive ρ‐response of the system.

Valency has a weaker and qualitatively different effect on the desorption side. Increasing κ initially shifts the desorption threshold $\rho_{\mathrm{dp}}$ to lower linker density, but this shift saturates rapidly at large κ (Figure 2d). The reason is that $\rho_{\mathrm{dp}}$ lies at high linker density, where it becomes entropically favorable to pack many linkers into the gap between the guest particle and the substrate. Accommodating each newly arriving linker forces those already bound to release some of their bonds. As a result, near desorption a bound linker typically forms only a few bonds, often just one, to a given surface, regardless of its nominal valency. The guest particle is instead held by many such linkers acting together, each contributing only a few bonds. Because this per‐linker contribution is limited by competition rather than set by κ, further increasing the valency adds little stabilization and has little effect on θ and $\rho_{\mathrm{dp}}$ [see Supporting Information]. This behavior is qualitatively consistent with the purely entropy‐driven phase behavior observed in programmable colloidal atom‐electron equivalents at large linker valency [17].

### 2.3 Detection Among Non‐Specific Binders

In complex environments such as biological fluids or environmental samples, numerous molecules other than the targeted linkers are present [24, 25, 26]. These untargeted species can adsorb onto sensor surfaces or recognition elements with weak non‐specific interactions. Generally, such non‐specific binding would introduce a background signal and reduce the selectivity and sensitivity of the detection system. Hence, careful consideration and mitigation of the environmental nonspecific binding is essential for developing a reliable detection system in real‐world applications. To this end, we generalize our model by considering non‐specific binders. Those non‐specific species are modeled as weak monovalent binders that can bond weakly with either ligands or receptors but cannot form bridges between them [27]. Within the present model, these non‐specific binders act competitively rather than cooperatively [22, 23]. Because they cannot bridge the two surfaces, they cannot contribute to bridge formation or lower the entropy cost paid by targeted linkers. Their role is to occupy ligands and receptors, reducing the pool of sites available for productive bridges. Here we only consider the non‐specific binders, as they are the most common environmental noise in bio‐detection, and the untargeted molecules bridging between ligands and receptors are rare [28]. Those binders are present in the environment with individual non‐specific binding free energies $f_{m}$ and treated as an ideal gas of density $\rho_{m}=e^{\beta \mu_{m}}/\Lambda^{3}$ with the chemical potential $\mu_{m}$.

We formulate the grand potential of the system with the mixture of the target linkers and non‐specific binders by applying the saddle‐point approximation. The resulting grand potential for the adsorbed state is [see Supporting Information]

(6) $$\beta F_{\mathrm{ad}}=\sum_{i\in \{l,r\}}\left(n_{i}\log{\bar{n}}_{i}-{\bar{n}}_{i}-M_{i}-B_{i}\right)-Q,$$

where $B_{i}$ is the number of non‐specific binders bound to ligands (i=l) or receptors (i=r). Likewise, for the desorbed state, where no bridge forms, the grand potential is

(7) $$\beta F_{\mathrm{dp}}=\sum_{i\in \{l,r\}}\left(n_{i}\log{\bar{n}}_{i}^{\prime }-{\bar{n}}_{i}^{\prime }-M_{i}^{\prime }-B_{i}^{\prime }\right),$$

where $B_{i}^{\prime }$ is the corresponding number of bound binders when the particle is far from the substrate.

**FIGURE 3:**
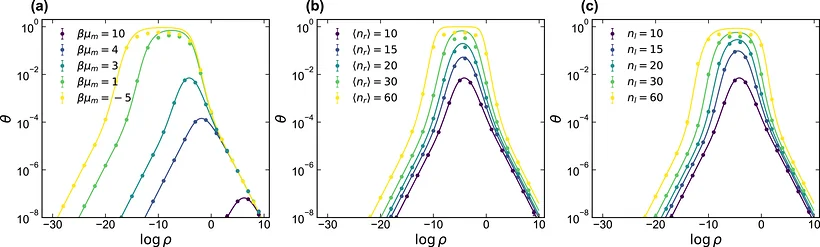
Influence of non‐specific binders. Parameters: $\kappa \beta f_{l}=\kappa \beta f_{r}=-5$, κ=3, $\beta f_{m}=1$, $\beta f_{\mathrm{cnf}}=2$, and $z_{g}=10^{-5}$. Symbols denote GCMC simulation results; solid curves are theoretical predictions from Equation (3). (a) Adsorption probability θ versus logρ for varying non‐specific binder chemical potential $\mu_{m}$, with $n_{l}=\left\langle n_{r}\right\rangle =10$. Increasing the binder concentration suppresses the adsorption plateau and shifts the detection threshold to higher linker densities. (b) Recovery of sensitivity by increasing the mean receptor number $\left\langle n_{r}\right\rangle$, with $n_{l}=10$ and $\beta \mu_{m}=3$. (c) Recovery of sensitivity by increasing the ligand number $n_{l}$ on guest particles, with $\langle n_{r}\rangle =10$ and $\beta \mu_{m}=3$. In both (b) and (c), increasing the number of binding sites restores the pool of available partners for targeted linkers, counteracting the competitive blocking by non‐specific binders.

The theoretical predictions and the simulation results for different chemical potential of binders are shown in Figure 3a. One can see that with increasing the chemical potential of the non‐specific binders, the adsorption of guest nanoparticles mediated by targeted linkers is significantly disrupted, and the saturation range gradually disappears with the detection threshold $\rho_{\mathrm{ad}}$ moving to a larger value, which indicates the vanishing of the ultrasensitivity. This suppression of linker‐mediated adsorption is due to the competition between non‐specific binders and targeted linkers for binding sites. By binding to ligands and receptors, the non‐specific binders effectively reduce the number of available binding partners for bridge formation. Therefore, a strategy to mitigate this effect is to increase the total number of ligands or receptors, thereby restoring available binding sites. As shown in Figure 3b,c, increasing the densities of receptors grafted on host substrate or the number of ligands coated on guest nanoparticles progressively recovers the detection sensitivity of targeted linkers. The reason is the same on either side: adding sites recruits more molecules into the contact region, including non‐specific binders and targeted linkers that dangle from the newly available sites. Combinatorial entropy keeps a mixture of dangling and bridging linker states populated. This larger pool of recruited linkers therefore also raises the number of bridges, rather than only adding dangling ones. Because the total number of bridges cannot exceed the number of sites on the scarcer side, this extra bridging pushes the scarcer side toward full bridge occupation. Increasing either $n_{l}$ or $\left\langle n_{r}\right\rangle$ therefore improves adsorption, even when the side being increased already has more sites than the other. Since both levers act through this same capping mechanism, neither is intrinsically more effective than the other: whichever side has fewer sites is the limiting factor. The recovery is nonetheless bounded, because once the scarcer side has nearly all its sites engaged in bridges, no further bridges can form and adding more sites no longer helps.

## 3 Discussion

In summary, we have developed a theoretical framework that elucidates how linker multivalency governs the sensitivity and selectivity of bio‐detection systems. Our analysis shows that, at a fixed overall binding strength, increasing linker valency exponentially lowers the model adsorption threshold $\rho_{\mathrm{ad}}$, pointing to ultrasensitive readouts at low target concentrations. In practical terms, sensitivity is governed by valency rather than by affinity: at fixed total binding strength, adding linker arms lowers the threshold through combinatorial entropy alone. Because this route is physical and needs no enzymatic replication, amplification‐free multivalent systems designed this way may narrow the sensitivity gap with PCR‐based assays. This is especially relevant for early‐stage biomarker and pathogen detection, where the target is scarce. At the same time, increasing linker valency reduces superselectivity with respect to linker concentration, reflecting a fundamental trade‐off between sensitivity and concentration‐based discrimination. Furthermore, the presence of non‐specific binders can suppress ultrasensitivity when their concentration becomes sufficiently high. This effect can be mitigated by adding binding sites, that is, by increasing the ligand number $n_{l}$ per particle or the mean receptor number $\left\langle n_{r}\right\rangle$ per site. Unlike the linker valency κ, this lever enlarges the pool of binding partners rather than redistributing a fixed binding strength, thereby restoring the detection sensitivity. Our results offer a physical explanation for the whole‐genome detection strategy of Xu et al. [14], where long ssDNA strands act as high‐valency linkers that drive colloidal aggregation at very low target concentrations. The many combinatorial binding configurations available to such linkers can lower the adsorption threshold without enzymatic amplification, which identifies entropy‐driven multivalent binding as a plausible origin of the observed sensitivity. This also points to design principles for linker‐mediated biosensors that combine high sensitivity with specificity in complex environments.

The framework also extends to environments containing several types of non‐specific binders. Because no binder can bridge the two surfaces, all of them add to the same competitive effect, occupying ligands and receptors that would otherwise host productive bridges. The more abundant, higher‐affinity, or higher‐valency species contribute most to this suppression. A multivalent binder, for example, engages several sites per molecule but still cannot bridge, so it acts only as a stronger competitor. The overall behavior therefore remains that of the single‐binder case. This is what distinguishes non‐specific binders from multivalent linkers: binders only compete for binding sites, whereas linkers bridge the two surfaces and promote adsorption.

Our coarse‐grained model leaves out several features of real linkers, including their length, flexibility, excluded volume, and spatial arrangement, and treats linkers as an ideal gas with mobile ligands. These simplifications mainly affect the local binding concentrations and configurational cost, and may shift the absolute thresholds and response kinetics. They leave the combinatorial entropy behind our central result intact, so the exponential lowering of $\rho_{\mathrm{ad}}$ with valency should remain robust. This threshold is a modeling convention: choosing $\theta_{\mathrm{th}}=0.01$, 0.1, or 0.2 gives a different $\rho_{\mathrm{ad}}$, and changing this choice only rescales it without altering the exponential scaling with valency. The practical limit of detection follows a different logic: it depends on whether the sensor can resolve a weak signal against background noise and readout sensitivity [29, 30, 31, 32]. These constraints grow more severe as the particle concentration decreases. Our model threshold thus describes how the onset of adsorption shifts with valency under a fixed definition, not the lowest concentration a real instrument can detect. Taken together, these findings indicate that optimal detection performance does not necessarily require ligands and receptors with the strongest possible binding affinities. Instead, those capable of forming multiple simultaneous interactions with the target may be more effective. These principles are directly applicable to rapid antigen tests, DNA‐mediated aggregation assays, and multivalent immunosensors, where signal generation relies on collective binding rather than enzymatic replication.

## 4 Methods

In this work, grand‐canonical Monte Carlo (GCMC) simulations are performed for 2D systems with implicit ligands and linkers. The guest particles are modeled as hard disks with radius σ/2, each coated with ligands. The ligands can interact with the receptors within σ/2 to the center of the guest particles. The receptors are distributed on the host substrate according to the Poisson distribution.

Due to the low density and the multivalent nature of guest particles in most experiments, the equilibration time for 3D simulations is extremely long. Therefore, we simulate a monolayer of guest particles on the substrate where the guest particles can be modeled as 2D hard disks, and they are in chemical equilibrium with a bulk 3D reservoir above. The adsorption and desorption of guest particles are treated as the insertion and deletion of particles from the reservoir in the simulation. The probabilities of attempting translational trial moves, particle addition trial moves, and deletion trial moves are set to 0.3, 0.35, and 0.35, respectively.

If a particle is inserted at a random position $s^{\prime }$, its effective interaction is described by the adsorption partition function $q\left(s^{\prime }\right)=e^{-\beta [F_{\mathrm{ad}}\left(s^{\prime }\right)-F_{\mathrm{dp}}\left(s^{\prime }\right)]}-1$. This trial move is accepted with probability

(8) $$\begin{array}{c} acc\left(N_{g}\to N_{g}+1\right)=\\ \min\left[1,\frac{Vq(s^{\prime })}{\Lambda^{2}\left(N_{g}+1\right)}e^{\beta [\mu_{g}-U\left(s^{\prime },N_{g}+1\right)+U\left(s^{\prime },N_{g}\right)]}\right], \end{array}$$

where V is the area of the 2D plane, Λ is the thermal de Broglie wavelength, $N_{g}$ is the number of guest particles before the insertion, $\beta \mu_{g}$ is the chemical potential of the reservoir of guest particles, and $U(s^{\prime },N_{g})$ quantifies the hard‐core repulsion. The deletion of a particle at position $s^{\prime }$ is accepted with probability

(9) $$\begin{array}{c} acc\left(N_{g}\to N_{g}-1\right)=\\ \min\left[1,\frac{\Lambda^{2}N_{g}}{Vq(s^{\prime })}e^{-\beta [\mu_{g}+U\left(s^{\prime },N_{g}-1\right)-U\left(s^{\prime },N_{g}\right)]}\right], \end{array}$$

where $N_{g}$ is the number of adsorbed guest particles before deletion. The translational trial move of a particle from a position s to $s^{\prime }$ is accepted with a probability

(10) $$acc\left(s\to s^{\prime }\right)=\min\left[1,\frac{q(s^{\prime })}{q(s)}e^{-\left(\beta U\left(s^{\prime },N_{g}\right)-\beta U\left(s,N_{g}\right)\right)}\right].$$

Moreover, periodic boundary conditions are applied in all directions. In each simulation, $5\times 10^{6}$ MC moves are performed for equilibration and $2\times 10^{7}$ MC moves for production, with sampling every 5 moves. The adsorption probability θ is computed as $N_{g}/N_{\max}$. Error bars in the figures represent the standard error over 20 independent realizations.

## Conflicts of Interest

The authors declare no conflicts of interests.

## Supporting information


**Supporting File**: advs77266‐sup‐0001‐SuppMat.pdf.

## Data Availability

The data that support the findings of this article are openly available at https://doi.org/10.5281/zenodo.21257813.
