# Supplementary material for: Entropy‐Driven Physical Amplification in Multivalent Biosensing
Source: Adv Sci (Weinh). 2026 Aug 25:e77266. Online ahead of print. doi: 10.1002/advs.77266 (PMC13505470; doi:10.1002/advs.77266)
Supplement: Supplementary file 1 [file ADVS-9999-e77266-s001.pdf]

Supplementary Material:
Entropy-driven physical amplification in multivalent biosensing

Yuhan Peng,[1,*] Xiuyang Xia *,[1,2,3,4,†] and Ran Ni[1,‡]

[1] *School of Chemistry, Chemical Engineering and Biotechnology, Nanyang Technological University, 62 Nanyang Drive, 637459, Singapore*

[2] *Arnold Sommerfeld Center for Theoretical Physics and Center for NanoScience, Department of Physics, Ludwig-Maximilians-Universität München, Theresienstraße 37, D-80333 München, Germany*

[3] *Department of Physics, Southern University of Science and Technology, Shenzhen, Guangdong 518055, China*

[4] *Center for Complex Flows and Soft Matter Research, Southern University of Science and Technology, Shenzhen, Guangdong 518055, China*

(Dated: July 6, 2026)

Contents

I. Mean-field theory for multivalent linker-mediated interaction 3

A. Without non-specific binders 3

1. Setup and notation 3
2. Adsorbed-state free energy 3
3. Self-consistent equations 4
4. Free energy in terms of saddle-point quantities 4
5. Desorbed-state free energy 5

B. With non-specific binders 5

1. Extended partition function 5
2. Saddle-point solution and self-consistent equations 5
3. Free energies with non-specific binders 6

II. Selectivity of linker-mediated multivalent adsorption 6

A. Derivatives of individual free-energy contributions 6

B. Implicit differentiation of the self-consistent equations 7

III. Behaviors of the system under different limits 7

A. Weak binding strength limit 8

B. Low linker density limit 8

C. High linker density limit 8

D. Maximal bridging limit (superselective regime) 9

1. Adsorption side ($\rho\xi_l^{\kappa_l} < 1$ and $\rho\xi_r^{\kappa_r} < 1$) 10
2. Desorption side ($\rho\xi_l^{\kappa_l} > 1$ and $\rho\xi_r^{\kappa_r} > 1$) 10

E. Large linker valency limit 11

1. Vanishing of linker populations 11
2. Entropy-dominated effective interaction 11
3. Adsorption threshold at large valency 12
4. Desorption threshold at large valency 12

IV. Adsorption and desorption thresholds with Poisson-distributed receptors 12

A. Inter-site relations between self-consistent solutions 13

B. Simplification in the large-valency limit 13

C. Determining the thresholds in the Poisson ensemble 13

*These authors contributed equally.

†Electronic address: `xiaxy@sustech.edu.cn`

‡Electronic address: `r.ni@ntu.edu.sg`

References 15

I. MEAN-FIELD THEORY FOR MULTIVALENT LINKER-MEDIATED INTERACTION

A. Without non-specific binders

1. Setup and notation

We consider a coarse-grained model in which guest nanoparticles are adsorbed onto a host substrate mediated by free multivalent linkers. Each site with a random number of receptors n_r on the host substrate can adsorb at most one guest particle coated with n_l ligands. Linkers are modeled as an ideal gas of chemical potential μ or bulk density $\rho = e^{\beta\mu}/\Lambda^3$ where Λ is the de Broglie wavelength, $\beta = 1/k_B T$, and with k_B and T being the Boltzmann constant and temperature of the system, respectively. Each multivalent linker has κ_l ends to bind with the ligands and its binding energy is f_l; likewise, each linker has κ_r ends to bind with the receptors and its binding energy is f_r. The formation of a bridge between a ligand and a receptor via a single linker incurs an additional mean-field configurational free-energy cost f_{cnf}.

In the adsorbed and desorbed states, a linker may exist in one of three classes:

1. *Dangling from ligands*: bound to i ligands on the guest particle ($i = 1, \ldots, \kappa_l$) but to no receptor. We denote the number of such linkers with i ligand bonds as m_l^i.
2. *Dangling from receptors*: bound to j receptors on the substrate ($j = 1, \ldots, \kappa_r$) but to no ligand. We denote their number as m_r^j.
3. *Bridging*: simultaneously bound to i ligands and j receptors, thereby connecting the guest particle to the substrate. We denote their number as b^{ij}. Bridging linkers are present only in the adsorbed state.

For notational compactness, we collect these into the sets $\mathbf{m}_l = \{m_l^1, \ldots, m_l^{\kappa_l}\}$, $\mathbf{m}_r = \{m_r^1, \ldots, m_r^{\kappa_r}\}$, and $\mathbf{b} = \{b^{ij}\}$ with $i = 1, \ldots, \kappa_l$ and $j = 1, \ldots, \kappa_r$.

2. Adsorbed-state free energy

In the adsorbed state, linkers from the solution can dangle from either surface or form bridges. The grand canonical partition function over all linker configurations reads

$$Z_{\mathrm{ad}}\left(\{\mathbf{m}_l, \mathbf{m}_r, \mathbf{b}\}\right) = \sum_{\{\mathbf{m}_l, \mathbf{m}_r, \mathbf{b}\}} W\left(\mathbf{m}_l, \mathbf{m}_r, \mathbf{b}\right) \prod_{i=1}^{\kappa_l} \left(e^{\beta\mu - i\beta f_l}\right)^{m_l^i} \prod_{j=1}^{\kappa_r} \left(e^{\beta\mu - j\beta f_r}\right)^{m_r^j} \prod_{i=1}^{\kappa_l} \prod_{j=1}^{\kappa_r} \left(e^{\beta\mu - \beta(i f_l + j f_r + f_{\mathrm{cnf}})}\right)^{b^{ij}}, \quad \text{(S1)}$$

where the three product terms account for the Boltzmann weights of linkers dangling from ligands, dangling from receptors, and bridging, respectively. The combinatorial prefactor $W(\mathbf{m}_l, \mathbf{m}_r, \mathbf{b})$ counts the number of distinct ways to assign linkers to binding sites and is given by [S1].

$$W\left(\mathbf{m}_l, \mathbf{m}_r, \mathbf{b}\right) = \frac{n_l! n_r!}{\bar{n}_l! \bar{n}_r!} \prod_{i}^{\kappa_l} \frac{\binom{\kappa_l}{i}^{m_l^i}}{m_l^i!} \prod_{j}^{\kappa_r} \frac{\binom{\kappa_r}{j}^{m_r^j}}{m_r^j!} \prod_{i,j} \frac{\left(\binom{\kappa_l}{i}\binom{\kappa_r}{j}\right)^{b^{ij}}}{b^{ij}!}. \quad \text{(S2)}$$

where $\bar{n}_l$ and $\bar{n}_r$ denote the numbers of unbound ligands and receptors in the adsorbed state.

We evaluate $\log Z_{\mathrm{ad}}$ using the saddle-point approximation, which yields the equilibrium (most probable) occupation numbers:

$$\begin{cases} m_l^i = (\bar{n}_l)^i \binom{\kappa_l}{i} e^{\beta\mu - i\beta f_l} \\ m_r^j = (\bar{n}_r)^j \binom{\kappa_r}{j} e^{\beta\mu - j\beta f_r} \\ b^{ij} = (\bar{n}_l)^i (\bar{n}_r)^j \binom{\kappa_l}{i} \binom{\kappa_r}{j} e^{\beta\mu - \beta(i f_l + j f_r + f_{\mathrm{cnf}})}, \end{cases} \quad \text{(S3)}$$

These expressions have an intuitive structure: each occupation number is proportional to a combinatorial factor (reflecting the number of ways to select binding ends), a Boltzmann weight (encoding the energetic cost), and a power of the number of available binding partners $\bar{n}_{l/r}$ (capturing the entropic multiplicity of target sites).

3. Self-consistent equations

The unbound numbers $\bar{n}_l$ and $\bar{n}_r$ are not free parameters but are determined self-consistently by the conservation of the total numbers of ligands and receptors:

$$\bar{n}_l + \sum_{i=1}^{\kappa_l} i m_l^i + \sum_{i=1}^{\kappa_l}\sum_{j=1}^{\kappa_r} i b^{ij} = n_l, \tag{S4}$$

$$\bar{n}_r + \sum_{i=1}^{\kappa_r} i m_r^i + \sum_{i=1}^{\kappa_l}\sum_{j=1}^{\kappa_r} j b^{ij} = n_r. \tag{S5}$$

Substituting the saddle-point solutions (S3) into Eqs. (S4) and (S5), and introducing the shorthand $\xi_l = e^{-\beta f_l}$ and $\xi_r = e^{-\beta f_r}$, the conservation laws reduce to two coupled nonlinear equations for $\bar{n}_l$ and $\bar{n}_r$:

$$\bar{n}_l + e^{\beta\mu}\kappa_l \bar{n}_l \xi_l \left(\bar{n}_l\xi_l + 1\right)^{\kappa_l - 1} + e^{\beta\mu - \beta f_{\rm cnf}}\kappa_l \bar{n}_l \xi_l \left(\bar{n}_l\xi_l + 1\right)^{\kappa_l - 1}\left[\left(\bar{n}_r\xi_r + 1\right)^{\kappa_r} - 1\right] = n_l, \tag{S6}$$

$$\bar{n}_r + e^{\beta\mu}\kappa_r \bar{n}_r \xi_r \left(\bar{n}_r\xi_r + 1\right)^{\kappa_r - 1} + e^{\beta\mu - \beta f_{\rm cnf}}\kappa_r \bar{n}_r \xi_r \left(\bar{n}_r\xi_r + 1\right)^{\kappa_r - 1}\left[\left(\bar{n}_l\xi_l + 1\right)^{\kappa_l} - 1\right] = n_r, \tag{S7}$$

Here, the three terms on the left-hand side of each equation correspond to unbound sites, sites occupied by dangling linkers, and sites occupied by bridging linkers, respectively. The key identities used in the reduction are

$$\sum_{i=1}^{\kappa_\gamma} i m_\gamma^i = e^{\beta\mu}\kappa_\gamma \bar{n}_\gamma \xi_\gamma \left(\bar{n}_\gamma \xi_\gamma + 1\right)^{\kappa_\gamma - 1} \tag{S8}$$

$$\sum_{i=1}^{\kappa_\gamma}\sum_{j=1}^{\kappa_\omega} i b^{ij} = e^{\beta\mu - \beta f_{\rm cnf}}\kappa_\gamma \bar{n}_\gamma \xi_\gamma \left(\bar{n}_\gamma \xi_\gamma + 1\right)^{\kappa_\gamma - 1}\left[\left(\bar{n}_\omega \xi_\omega + 1\right)^{\kappa_\omega} - 1\right] \tag{S9}$$

where $\gamma \neq \omega \in \{l, r\}$, $\xi_l = e^{-\beta f_l}$, and $\xi_r = e^{-\beta f_r}$. Unlike quadratic equations in previous studies [S2], the higher-order equations above have no closed-form solutions, and we solve them numerically using fixed-point iteration throughout this work.

4. Free energy in terms of saddle-point quantities

Substituting the saddle-point solution back into $\beta F_{\rm ad} = -\log Z_{\rm ad}$ evaluated at the most probable configuration, we obtain

$$\beta F_{\rm ad} = \sum_{\gamma\in\{l,r\}}\left[n_\gamma\left(\log\frac{\bar{n}_\gamma}{n_\gamma} + 1\right) - \bar{n}_\gamma - \sum_{k=1}^{\kappa_\gamma} m_\gamma^i\right] - \sum_{\substack{i=1\\j=1}}^{\kappa_l \kappa_r} b^{ij}. \tag{S10}$$

where the total numbers of dangling and bridging linkers appearing in Eq. (S10) are conveniently expressed as

$$\sum_{i=1}^{\kappa_\gamma} m_\gamma^i = e^{\beta\mu}\left[\left(\bar{n}_\gamma\xi_\gamma + 1\right)^{\kappa_\gamma} - 1\right], \tag{S11}$$

$$\sum_{\substack{i=1\\j=1}}^{\kappa_l \kappa_r} b^{ij} = e^{\beta\mu - \beta f_{\rm cnf}}\left[\left(\bar{n}_l\xi_l + 1\right)^{\kappa_l} - 1\right]\left[\left(\bar{n}_r\xi_r + 1\right)^{\kappa_r} - 1\right]. \tag{S12}$$

5. Desorbed-state free energy

In the desorbed state, the guest particle is infinitely far from the substrate site, so no bridge can form. The partition function therefore involves only dangling linkers:

$$Z'(\mathbf{m}'_l, \mathbf{m}'_r) = \sum_{\{\mathbf{m}'_l, \mathbf{m}'_r\}} W(\mathbf{m}'_l, \mathbf{m}'_r) \prod_{i=1}^{\kappa_l} (e^{\beta\mu - i\beta f_l})^{m_l'^{(i)}} \prod_{j=1}^{\kappa_r} (e^{\beta\mu - j\beta f_r})^{m_r'^{(j)}}, \tag{S13}$$

where primed quantities denote the desorbed-state counterparts. The combinatorial weight takes the same form as Eq. (S2) but with the bridging sector removed:

$$W(\mathbf{m}'_l, \mathbf{m}'_r) = \frac{n_l!\, n_r!}{\bar{n}'_l!\, \bar{n}'_r!} \prod_i \frac{\binom{\kappa_l}{i}^{m_l'^{(i)}}}{m_l'^{(i)}!} \prod_j \frac{\binom{\kappa_r}{j}^{m_r'^{(j)}}}{m_r'^{(j)}!}. \tag{S14}$$

Following the same saddle-point procedure, the desorbed-state free energy is

$$\beta F_{\mathrm{dp}} = \sum_{\gamma \in \{l,r\}} \left[n_\gamma \left(\log \frac{\bar{n}'_\gamma}{n_\gamma} + 1 \right) - \bar{n}'_\gamma - \sum_{k=1}^{\kappa_\gamma} m_\gamma'^{(k)} \right], \tag{S15}$$

where $\bar{n}'_\gamma$ denotes the number of unbound ligands ($\gamma = l$) or receptors ($\gamma = r$) in the desorbed state. The self-consistent equations for $\bar{n}'_l$ and $\bar{n}'_r$ are obtained from Eqs. (S4)–(S5) by removing the bridging terms (equivalently, taking $f_{\mathrm{cnf}} \to \infty$).

B. With non-specific binders

We now generalize the framework to include non-specific binders—monovalent molecules present in the environment that can bind weakly to either ligands or receptors but cannot form bridges between them. Such species are the dominant source of background noise in most biodetection settings. We model them as an ideal gas with chemical potential μ_m and individual binding free energy f_m to either ligands or receptors.

1. Extended partition function

The grand canonical partition function is extended to include binder occupation numbers E_{al} (binders adsorbed onto ligands) and E_{ar} (binders adsorbed onto receptors):

$$Z_{\mathrm{ad}}(\{\mathbf{m}_l, \mathbf{m}_r, \mathbf{b}, E_{al}, E_{ar}\}) = \sum_{\{\mathbf{m}_l, \mathbf{m}_r, \mathbf{b}\}} W(\mathbf{m}_l, \mathbf{m}_r, \mathbf{b}, E_{al}, E_{ar}) \prod_{i=1}^{\kappa_l} \left(e^{\beta\mu - i\beta f_l}\right)^{m_l^i} \prod_{j=1}^{\kappa_r} \left(e^{\beta\mu - j\beta f_r}\right)^{m_r^j} \prod_{i=1}^{\kappa_l} \prod_{j=1}^{\kappa_r} \left(e^{\beta\mu - \beta(i f_l + j f_r + f_{\mathrm{cnf}})}\right)^{b^{ij}} \left(e^{\beta\mu_m - \beta f_m}\right)^{E_{al} + E_{ar}}, \tag{S16}$$

where the combinatorial weight is modified to account for the additional partitioning of binding sites among linkers and binders:

$$W(\mathbf{m}_l, \mathbf{m}_r, \mathbf{b}, E_{al}, E_{ar}) = \frac{n_l! n_r!}{\bar{n}_l! \bar{n}_r! E_{al}! E_{ar}!} \prod_i^{\kappa_l} \frac{\binom{\kappa_l}{i}^{m_l^i}}{m_l^i!} \prod_j^{\kappa_r} \frac{\binom{\kappa_r}{j}^{m_r^j}}{m_r^j!} \prod_{i,j} \frac{\left(\binom{\kappa_l}{i}\binom{\kappa_r}{j}\right)^{b^{ij}}}{b^{ij}!}. \tag{S17}$$

2. Saddle-point solution and self-consistent equations

The saddle-point approximation yields the same linker occupation numbers as Eq. (S3), supplemented by the equilibrium binder numbers:

$$E_{al} = \bar{n}_l \, e^{\beta\mu_m - \beta f_m}, \qquad E_{ar} = \bar{n}_r \, e^{\beta\mu_m - \beta f_m}. \tag{S18}$$

The self-consistent equations are modified by including the binder contributions in the conservation laws:

$$\bar{n}_l + \sum_i i \, m_l^i + \sum_{i,j} i \, b^{ij} + E_{al} = n_l, \tag{S19}$$

$$\bar{n}_r + \sum_j j \, m_r^j + \sum_{i,j} j \, b^{ij} + E_{ar} = n_r. \tag{S20}$$

Compared with Eqs. (S4) and (S5), the only change is the addition of the binder terms $E_{a\gamma}$, which effectively reduce the pool of available binding partners for the targeted linkers.

3. Free energies with non-specific binders

The adsorbed-state free energy generalizes to

$$\beta F_{\rm ad} = \sum_{\gamma \in \{l,r\}} \left[n_\gamma \left(\log \frac{\bar{n}_\gamma}{n_\gamma} + 1 \right) - \bar{n}_\gamma - \sum_k m_\gamma^k - B_\gamma \right] - \sum_{i,j} b^{ij} - E_{al} - E_{ar}, \tag{S21}$$

and the desorbed-state free energy becomes

$$\beta F_{\rm dp} = \sum_{\gamma \in \{l,r\}} \left[n_\gamma \left(\log \frac{\bar{n}'_\gamma}{n_\gamma} + 1 \right) - \bar{n}'_\gamma - \sum_k m_\gamma'^{(k)} - B'_\gamma \right], \tag{S22}$$

where B'_γ is the number of adsorbed binders in the desorbed state. As before, the desorbed-state quantities are obtained by taking $f_{\rm cnf} \to \infty$, which eliminates all bridging terms while retaining the competition between dangling linkers and non-specific binders for the available binding sites.

II. SELECTIVITY OF LINKER-MEDIATED MULTIVALENT ADSORPTION

The selectivity parameter $\alpha_\chi = d\log\theta / d\log\chi$ quantifies how sensitively the adsorption probability θ responds to changes in an environmental parameter χ (e.g., linker density ρ, binding strength, or receptor number). In this section, we derive a general expression for α_χ in terms of the equilibrium unbound numbers $\bar{n}_l$ and $\bar{n}_r$, which are themselves implicit functions of the system parameters through the self-consistent equations derived in Sec. I.

$$\alpha_\chi = \frac{\mathrm{dlog}\theta}{\mathrm{dlog}\chi} = \frac{\partial \mathrm{log}\theta}{\partial F_{\rm eff}} \frac{\mathrm{d}F_{\rm eff}}{\mathrm{dlog}\chi} + \frac{\partial \mathrm{log}\theta}{\partial \mathrm{log}\chi}, \tag{S23}$$

where $F_{\rm eff} = F_{\rm ad} - F_{\rm dp}$ is the effective interaction free energy. The first term captures the indirect dependence of θ on χ through the effective interaction, while the second accounts for any explicit dependence (e.g., when χ appears directly in the Langmuir isotherm, as is the case for the guest particle activity z_g).

The key task is therefore to evaluate $dF_{\rm eff}/d\log\chi$, which requires tracking how the equilibrium unbound numbers $\bar{n}_l$ and $\bar{n}_r$ respond to changes in χ. Below, we express each contribution to this derivative in terms of $d\bar{n}_\gamma / d\log\chi$ (with $\gamma \in \{l, r\}$).

A. Derivatives of individual free-energy contributions

The effective interaction $F_{\rm eff}$ consists of three types of terms: the unbound-site entropy, the dangling-linker contributions, and the bridging-linker contributions. We differentiate each in turn.

a. Unbound-site entropy. The unbound-site terms contribute

$$\frac{d}{d\log\chi} \sum_\gamma n_\gamma \left(\log \frac{\bar{n}_\gamma}{n_\gamma} + 1 \right) - \bar{n}_\gamma = \sum_\gamma \left(\frac{n_\gamma}{\bar{n}_\gamma} - 1 \right) \frac{d\bar{n}_\gamma}{d\log\chi}\,. \tag{S24}$$

b. Dangling-linker terms. The total number of dangling linkers on species γ satisfies $\sum_i i\, m_\gamma^i = e^{\beta\mu}\, \kappa_\gamma \bar{n}_\gamma \xi_\gamma (\bar{n}_\gamma \xi_\gamma + 1)^{\kappa_\gamma - 1}$ [Eq. (S8)], and its derivative is

$$\frac{\mathrm{d}}{\mathrm{d}\log\chi} \sum_i m_\gamma^i = \frac{\partial}{\partial \log\chi} \sum_i m_\gamma^i + \sum_\gamma e^{\beta\mu} \kappa_\gamma \xi_\gamma \left(\bar{n}_\gamma \xi_\gamma + 1\right)^{\kappa_\gamma - 1} \frac{\mathrm{d}\bar{\mathrm{n}}_\gamma}{\mathrm{d}\log\chi}, \tag{S25}$$

where the first (partial) term captures the explicit dependence on χ through parameters such as μ or ξ_γ, and the second term arises from the implicit dependence through $\bar{n}_\gamma$.

c. Bridging-linker terms. Similarly, the total number of bridging linker bonds contributed by species γ satisfies $\sum_{i,j} i\, b^{ij} = e^{\beta\mu - \beta f_{\mathrm{cnf}}}\, \kappa_\gamma \bar{n}_\gamma \xi_\gamma (\bar{n}_\gamma \xi_\gamma + 1)^{\kappa_\gamma - 1}[(\bar{n}_\omega \xi_\omega + 1)^{\kappa_\omega} - 1]$ [Eq. (S9)], giving

$$\frac{\mathrm{d}}{\mathrm{d}\log\chi} \sum_{i,j}^{\kappa_l,\kappa_r} b^{ij} = \frac{\partial}{\partial \log\chi} \sum_{i,j}^{\kappa_l,\kappa_r} b^{ij} + \sum_{\gamma \neq \omega \in \{l,r\}} e^{\beta\mu - \beta f_{\mathrm{cnf}}} \kappa_\gamma \xi_\gamma \left(\bar{n}_\gamma \xi_\gamma + 1\right)^{\kappa_\gamma - 1} \left[(\bar{n}_\omega \xi_\omega + 1)^{\kappa_\omega} - 1\right] \frac{\mathrm{d}\bar{\mathrm{n}}_\gamma}{\log\chi}. \tag{S26}$$

B. Implicit differentiation of the self-consistent equations

The remaining unknowns, $d\bar{n}_l/d\log\chi$ and $d\bar{n}_r/d\log\chi$, are determined by implicit differentiation of the self-consistent equations. To this end, we define

$$G_l(\bar{n}_l, \bar{n}_r; \beta\mu, \xi_l, \xi_r, \kappa_r, \kappa_l) = \bar{n}_l + e^{\beta\mu} \kappa_l \bar{n}_l \xi_l \left(\bar{n}_l \xi_l + 1\right)^{\kappa_l - 1} + e^{\beta\mu - \beta f_{\mathrm{cnf}}} \kappa_l \bar{n}_l \xi_l \left(\bar{n}_l \xi_l + 1\right)^{\kappa_l - 1} \left[(\bar{n}_r \xi_r + 1)^{\kappa_r} - 1\right] - n_l, \tag{S27}$$

$$G_r(\bar{n}_l, \bar{n}_r; \beta\mu, \xi_l, \xi_r, \kappa_r, \kappa_l) = \bar{n}_r + e^{\beta\mu} \kappa_r \bar{n}_r \xi_r \left(\bar{n}_r \xi_r + 1\right)^{\kappa_r - 1} + e^{\beta\mu - \beta f_{\mathrm{cnf}}} \kappa_r \bar{n}_r \xi_r \left(\bar{n}_r \xi_r + 1\right)^{\kappa_r - 1} \left[(\bar{n}_l \xi_l + 1)^{\kappa_l} - 1\right] - n_r, \tag{S28}$$

so that the self-consistent equations are expressed compactly as $G_l(\bar{n}_l, \bar{n}_r; \beta\mu, \xi_l, \xi_r, \kappa_r, \kappa_l) = 0$ and $G_r(\bar{n}_l, \bar{n}_r; \beta\mu, \xi_l, \xi_r, \kappa_r, \kappa_l) = 0$, where $\bar{n}_l$ and $\bar{n}_r$ are implicit functions of the external parameters. Total differentiation of these constraints with respect to $\log\chi$ yields a 2×2 linear system:

$$\frac{\partial G_l}{\partial \bar{n}_l} \frac{\mathrm{d}\bar{n}_l}{\mathrm{d}\log\chi} + \frac{\partial G_l}{\partial \bar{n}_r} \frac{\mathrm{d}\bar{n}_r}{\mathrm{d}\log\chi} = -\frac{\partial G_l}{\partial \log\chi}, \tag{S29}$$

$$\frac{\partial G_r}{\partial \bar{n}_l} \frac{\mathrm{d}\bar{n}_l}{\mathrm{d}\log\chi} + \frac{\partial G_r}{\partial \bar{n}_r} \frac{\mathrm{d}\bar{n}_r}{\mathrm{d}\log\chi} = -\frac{\partial G_r}{\partial \log\chi}. \tag{S30}$$

Solving this linear system provides $d\bar{n}_l/d\log\chi$ and $d\bar{n}_r/d\log\chi$ for any choice of the parameter χ. Together with Eqs. (S24)–(S26), this fully determines the selectivity α_χ via Eq. (S23).

We note that while the expressions above are derived for the system without non-specific binders, the extension to include binders follows the same structure: the functions G_l and G_r acquire additional binder terms [Eqs. (S19)–(S20)], and the implicit differentiation proceeds identically.

III. BEHAVIORS OF THE SYSTEM UNDER DIFFERENT LIMITS

The self-consistent equations derived in Sec. I must in general be solved numerically. However, considerable physical insight can be gained by examining analytically tractable limits. In this section, we study five such regimes, ordered by increasing physical complexity:

1. **Weak binding strength** ($\xi_l, \xi_r \to 0$): individual bonds are too weak to appreciably deplete unbound sites (Sec. III A).
2. **Low linker density** ($\rho = e^{\beta\mu} \to 0$): linkers are sparse and most binding sites remain unoccupied (Sec. III B).
3. **High linker density** ($\rho \to \infty$): nearly all binding sites are saturated by linkers (Sec. III C).
4. **Maximal bridging** (strong binding at moderate density): the superselective regime in which bridging linkers dominate (Sec. III D).
5. **Large linker valency** ($\kappa \to \infty$ at fixed total binding strength): the regime most relevant to the main text, where the exponential scaling of the adsorption threshold with valency emerges (Sec. III E).

In each case, we derive the leading-order scaling of the adsorption probability θ and, where relevant, the selectivity α_ρ. A summary of the key scalings is collected in Table I at the end of this section.

A. Weak binding strength limit

We first consider the regime where individual binding is weak, i.e., $\xi_\gamma = e^{-\beta f_\gamma} \to 0$ for $\gamma \in \{l, r\}$. Physically, this means that even in the presence of linkers, very few bonds are formed, and the pool of available binding sites is barely depleted.

a. Unbound numbers. Expanding the self-consistent equations (S4)–(S5) to leading order in ξ_l and ξ_r using $(1+x)^\kappa \approx 1 + \kappa x$ for $x \to 0$, the adsorbed-state equations become

$$\bar{n}_l + e^{\beta\mu}\kappa_l \bar{n}_l \xi_l \big[1 + (\kappa_l - 1)\bar{n}_l \xi_l\big]\big[1 + e^{-\beta f_{\text{cnf}}}\kappa_r \bar{n}_r \xi_r\big] = n_l \,, \tag{S31}$$

$$\bar{n}_r + e^{\beta\mu}\kappa_r \bar{n}_r \xi_r \big[1 + (\kappa_r - 1)\bar{n}_r \xi_r\big]\big[1 + e^{-\beta f_{\text{cnf}}}\kappa_l \bar{n}_l \xi_l\big] = n_r \,. \tag{S32}$$

The desorbed-state equations take the same form without the bridging factor (the term involving f_{cnf}). At leading order, both states yield the same solution:

$$\bar{n}_l \approx \bar{n}'_l \approx \frac{n_l}{1 + \kappa_l e^{\beta\mu}\xi_l} \approx n_l \,, \qquad \bar{n}_r \approx \bar{n}'_r \approx \frac{n_r}{1 + \kappa_r e^{\beta\mu}\xi_r} \approx n_r \,. \tag{S33}$$

That is, the binding is so weak that essentially all ligands and receptors remain unbound.

b. Effective interaction and adsorption probability. Since $\bar{n}_\gamma \approx \bar{n}'_\gamma$, the unbound-site entropy terms cancel between the adsorbed and desorbed states, and the effective interaction is dominated by the bridging contribution:

$$\beta F_{\text{eff}} \approx -\sum_{i,j} b^{(ij)} \approx -n_l n_r \, e^{\beta\mu - \beta f_{\text{cnf}}} \, \xi_l \xi_r \, \kappa_l \kappa_r \,. \tag{S34}$$

The adsorption probability in this regime is therefore

$$\theta \approx z_g q \approx z_g(-\beta F_{\text{eff}}) = z_g \, n_l n_r \, e^{\beta\mu - \beta f_{\text{cnf}}} \, \xi_l \xi_r \, \kappa_l \kappa_r \,, \tag{S35}$$

which scales linearly with both binding partition functions: $\theta \sim \xi_l \, \xi_r$. This linear scaling reflects the fact that, in the weak-binding regime, each linker forms at most one bond with each surface, and bonds are too rare to interact cooperatively.

B. Low linker density limit

Next, we consider the regime of vanishingly low linker density, $\rho = e^{\beta\mu} \to 0$, while keeping the binding strengths ξ_l and ξ_r arbitrary. In this limit, linkers are too scarce to appreciably occupy any binding sites, regardless of how strong individual bonds may be.

a. Unbound numbers. Retaining only the leading-order terms in ρ in the self-consistent equations, we find

$$\bar{n}_l = n_l - e^{\beta\mu}\kappa_l n_l \xi_l (n_l \xi_l + 1)^{\kappa_l - 1}\big\{1 + e^{-\beta f_{\text{cnf}}}\big[(n_r \xi_r + 1)^{\kappa_r} - 1\big]\big\} \approx n_l \,, \tag{S36}$$

$$\bar{n}_r = n_r - e^{\beta\mu}\kappa_r n_r \xi_r (n_r \xi_r + 1)^{\kappa_r - 1}\big\{1 + e^{-\beta f_{\text{cnf}}}\big[(n_l \xi_l + 1)^{\kappa_l} - 1\big]\big\} \approx n_r \,, \tag{S37}$$

with analogous expressions for the desorbed state: $\bar{n}'_l \approx n_l$ and $\bar{n}'_r \approx n_r$.

b. Effective interaction and adsorption probability. As in the weak-binding case, the unbound-site terms cancel between adsorbed and desorbed states, and the effective interaction reduces to the bridging contribution—but now evaluated at full binding strength:

$$\beta F_{\text{eff}} \approx -e^{\beta\mu - \beta f_{\text{cnf}}}\big[(n_l \xi_l + 1)^{\kappa_l} - 1\big]\big[(n_r \xi_r + 1)^{\kappa_r} - 1\big]. \tag{S38}$$

The adsorption probability is therefore

$$\theta \approx z_g \, e^{\beta\mu - \beta f_{\text{cnf}}}\big[(n_l \xi_l + 1)^{\kappa_l} - 1\big]\big[(n_r \xi_r + 1)^{\kappa_r} - 1\big], \tag{S39}$$

which scales linearly with the linker density: $\theta \sim \rho$. This is the expected behavior at the onset of adsorption, where each additional linker independently contributes to bridging without competition for binding sites.

C. High linker density limit

At high linker density ($\rho = e^{\beta\mu} \to \infty$), nearly all ligands and receptors are bound by linkers, so that $\bar{n}_\gamma \to 0$. This regime corresponds to the desorption side of the adsorption curve, where an excess of linkers saturates both surfaces and suppresses productive bridging.

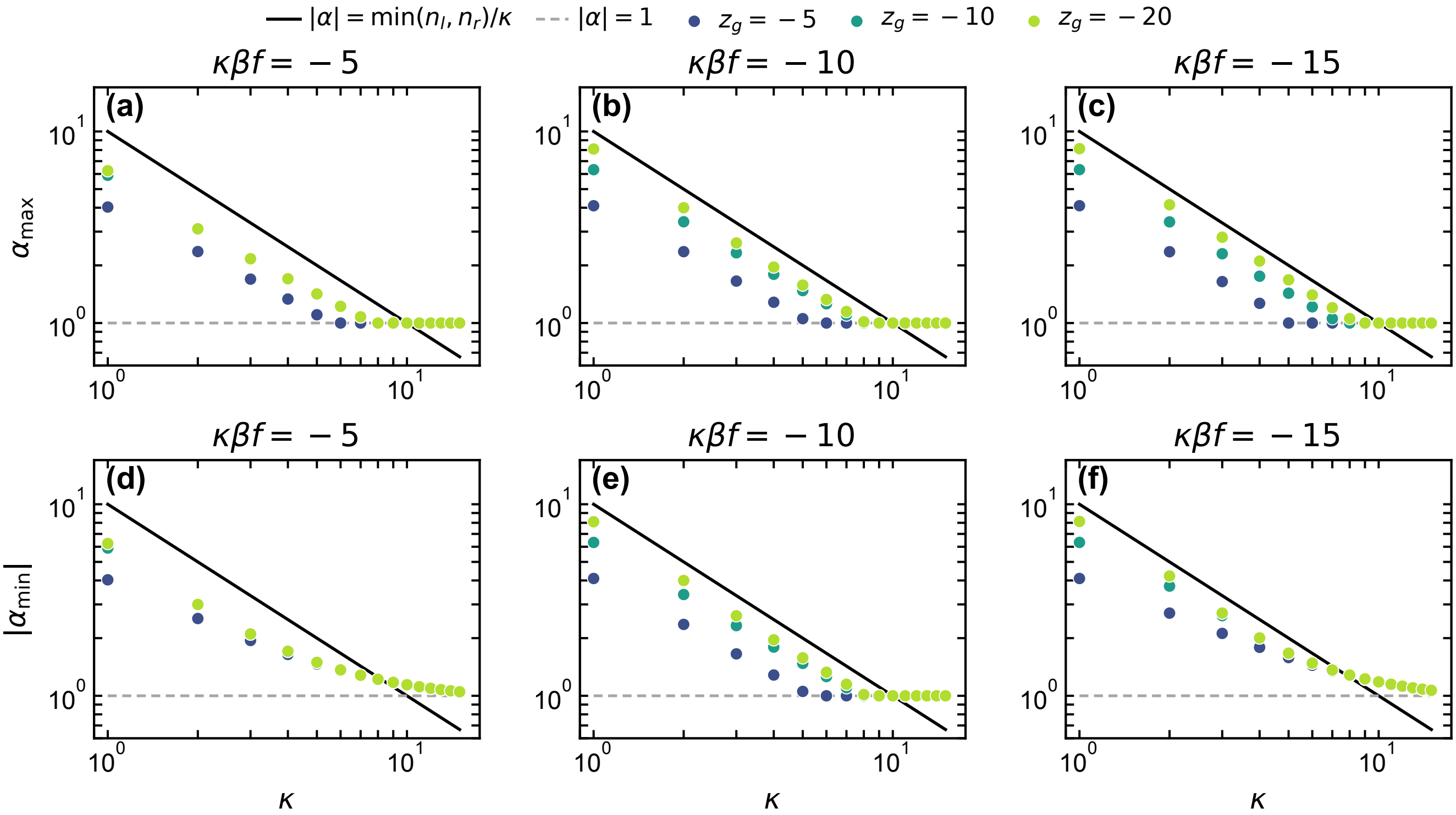

FIG. S1: **Relation between selectivity and valency**. Parameters: $\beta f_l = \beta f_r = \beta f$, $n_l = \langle n_r \rangle = 10$, and $\beta f_{\rm cnf} = 2$. Symbols show results from the full mean-field theory; solid lines show the large-κ asymptotic prediction $|\alpha_\rho| = \min(n_l, n_r)/\kappa$; dashed lines mark $|\alpha| = 1$. Top row [(a)–(c)]: maximum selectivity $\alpha_{\rm max}$ (adsorption side) versus κ. Bottom row [(d)–(f)]: magnitude of minimum selectivity $|\alpha_{\rm min}|$ (desorption side) versus κ. Columns correspond to different fixed total binding strengths: $\kappa\beta f = -5$ [(a),(d)], -10 [(b),(e)], and -15 [(c),(f)]. Different colors represent guest particle activities $z_g = 10^{-5}$, 10^{-10}, and 10^{-20}. The asymptotic scaling is approached more closely at lower z_g and stronger binding, consistent with the idealized limits assumed in the derivation (Sec. III D)

a. Unbound numbers. Expanding the self-consistent equations to leading order in $\bar{n}_\gamma \to 0$ using $(1+x)^\kappa \approx 1+\kappa x$, the adsorbed- and desorbed-state equations both yield

$$\bar{n}_l = \bar{n}_l' \approx \frac{n_l}{\kappa_l e^{\beta\mu}\xi_l}\,, \qquad \bar{n}_r = \bar{n}_r' \approx \frac{n_r}{\kappa_r e^{\beta\mu}\xi_r}\,. \tag{S40}$$

The unbound numbers decrease as $\bar{n}_\gamma \sim \rho^{-1}$, confirming that binding sites are progressively saturated.

b. Effective interaction and adsorption probability. Substituting Eq. (S40) into the bridging term, the effective interaction becomes

$$\beta F_{\rm eff} \approx -e^{\beta\mu - \beta f_{\rm cnf}}\xi_l\xi_r\kappa_l\kappa_r\bar{n}_l\bar{n}_r = -e^{-\beta f_{\rm cnf} - \beta\mu}\, n_l n_r\,, \tag{S41}$$

and the adsorption probability scales as

$$\theta \approx e^{-\beta f_{\rm cnf}}\, n_l n_r\, \rho^{-1}\,, \tag{S42}$$

i.e., $\theta \sim \rho^{-1}$. The inverse scaling with density reflects the fact that excess linkers saturate binding sites without forming productive bridges—the hallmark of the "hook effect" characteristic of linker-mediated systems [S1].

D. Maximal bridging limit (superselective regime)

Between the low-density onset ($\theta \sim \rho$) and the high-density decay ($\theta \sim \rho^{-1}$), there exists an intermediate regime in which virtually all bound linkers form bridges rather than dangling. This "maximal bridging" regime gives rise to the superselective response observed in Fig. 2 of the main text. To isolate this behavior analytically, we consider the idealized limit $\rho \to 0$ and $\xi_{l/r} \to \infty$ with $\rho\,\xi_\gamma^{\kappa_\gamma}$ held at moderate values, and take $z_g \to 0$ so that $\theta \ll 1$.

We treat the adsorption and desorption sides of this regime separately, as they yield distinct scaling behaviors.

1. *Adsorption side ($\rho\xi_l^{\kappa_l} < 1$ and $\rho\xi_r^{\kappa_r} < 1$)*

On the adsorption side of the superselective regime, binding is strong enough that all linkers bridge, yet linkers are still sufficiently sparse that some binding sites remain unoccupied. We assume without loss of generality that $\kappa_l/n_l > \kappa_r/n_r$, meaning that ligands become fully bound before receptors.

Using $(1+x)^\kappa \approx x^\kappa$ for $x \gg 1$, and noting that ligands are nearly fully bound while receptors retain a finite number of unbound sites, the self-consistent equations reduce to

$$\kappa_l \, e^{\beta\mu - \beta f_{\rm cnf}} \, \bar{n}_l^{\kappa_l} \bar{n}_r^{\kappa_r} \, \xi_l^{\kappa_l} \xi_r^{\kappa_r} = n_l \,, \tag{S43}$$

$$\bar{n}_r + \kappa_r \, e^{\beta\mu - \beta f_{\rm cnf}} \, \bar{n}_l^{\kappa_l} \bar{n}_r^{\kappa_r} \, \xi_l^{\kappa_l} \xi_r^{\kappa_r} = n_r \,, \tag{S44}$$

where dangling-linker terms have been neglected relative to bridging terms. The solutions are

$$\bar{n}_l \approx \left(\frac{n_l}{\kappa_l \, e^{\beta\mu - \beta f_{\rm cnf}} \, \bar{n}_r^{\kappa_r} \, \xi_l^{\kappa_l} \xi_r^{\kappa_r}} \right)^{1/\kappa_l} , \qquad \bar{n}_r \approx n_r - \frac{\kappa_r}{\kappa_l} n_l \,. \tag{S45}$$

In the desorbed state, linkers are too sparse to occupy significant numbers of sites ($\rho \to 0$), so $\bar{n}_l' \approx n_l$ and $\bar{n}_r' \approx n_r$. The dominant contribution to the effective interaction is therefore $n_l \log(\bar{n}_l/n_l)$, and the adsorption probability scales as

$$\theta \approx z_g \left[\kappa_l \, e^{\beta\mu - \beta f_{\rm cnf}} \, (n_r - \kappa_r n_l/\kappa_l)^{\kappa_r} \, \xi_l^{\kappa_l} \xi_r^{\kappa_r} \right]^{n_l/\kappa_l} . \tag{S46}$$

This expression shows that the maximal selectivity with respect to linker density in this regime is $\alpha_\rho \sim n_l/\kappa_l$ (See Fig. S1), while the selectivity with respect to the binding partition functions $\xi_{l/r}$ is unaffected by valency. More generally, when $\kappa_l/n_l < \kappa_r/n_r$, the roles of ligands and receptors are interchanged and $\alpha_\rho \sim n_r/\kappa_r$, so that

$$\alpha_{\rho,\max} \sim \frac{\min(n_l, n_r)}{\kappa} \,, \tag{S47}$$

under the symmetric protocol $\kappa_l = \kappa_r = \kappa$ used in the main text.

2. *Desorption side ($\rho\xi_l^{\kappa_l} > 1$ and $\rho\xi_r^{\kappa_r} > 1$)*

On the desorption side, binding is sufficiently strong that both ligands and receptors are nearly fully occupied. Again assuming $\kappa_l/n_l > \kappa_r/n_r$, the self-consistent equations for the adsorbed state become

$$\kappa_l \, e^{\beta\mu - \beta f_{\rm cnf}} \, \bar{n}_l^{\kappa_l} \bar{n}_r^{\kappa_r} \, \xi_l^{\kappa_l} \xi_r^{\kappa_r} = n_l \,, \tag{S48}$$

$$\kappa_r \, e^{\beta\mu} \, \bar{n}_r^{\kappa_r} \, \xi_r^{\kappa_r} + \kappa_r \, e^{\beta\mu - \beta f_{\rm cnf}} \, \bar{n}_l^{\kappa_l} \bar{n}_r^{\kappa_r} \, \xi_l^{\kappa_l} \xi_r^{\kappa_r} = n_r \,, \tag{S49}$$

where the first term in Eq. (S49) accounts for linkers dangling from receptors, which become significant when receptors are saturated. The solutions are

$$\bar{n}_l = \left(\frac{n_l}{\kappa_l e^{-\beta f_{\rm cnf}} (n_r - \kappa_r n_l/\kappa_l) \, \xi_l^{\kappa_l}} \right)^{1/\kappa_l} , \qquad \bar{n}_r = \left(\frac{n_r - \kappa_r n_l/\kappa_l}{e^{\beta\mu} \kappa_r \xi_r^{\kappa_r}} \right)^{1/\kappa_r} . \tag{S50}$$

In the desorbed state, binding is extremely strong, so the unbound numbers scale as $\bar{n}_\gamma' \approx (n_\gamma/\kappa_\gamma e^{\beta\mu} \xi_\gamma^{\kappa_\gamma})^{1/\kappa_\gamma}$. The effective interaction is dominated by

$$\beta F_{\rm eff} \approx \frac{n_l}{\kappa_l} \log \frac{e^{\beta\mu}}{e^{-\beta f_{\rm cnf}} (n_r - \kappa_r n_l/\kappa_l)} + \frac{n_r}{\kappa_r} \log \frac{n_r - \kappa_r n_l/\kappa_l}{n_r} \,, \tag{S51}$$

yielding

$$\theta \approx z_g \left(\frac{e^{-\beta f_{\rm cnf}} (n_r - n_l)}{e^{\beta\mu}} \right)^{n_l/\kappa_l} . \tag{S52}$$

The selectivity on the desorption side is therefore $\alpha_\rho \sim -n_l/\kappa_l$(See Fig. S1), or more generally $\alpha_{\rho,\min} \sim -\min(n_l, n_r)/\kappa$ under the symmetric protocol. Fig. S1 confirms that these scaling predictions agree quantitatively with the full mean-field theory as $z_g \to 0$ and $\xi_{l/r} \to \infty$.

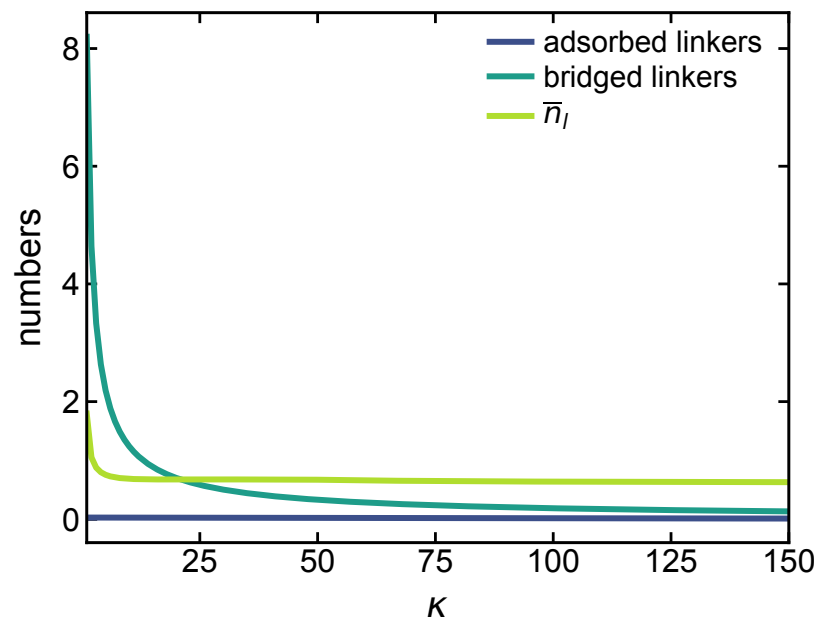

FIG. S2: **Vanishing of linker populations in the large-valency limit.** Parameters: $\kappa\beta f_l = \kappa\beta f_r = -5$, $n_l = \langle n_r \rangle = 10$, $\beta f_{\rm cnf} = 2$, and $z_g = 10^{-5}$. The total numbers of dangling linkers (dark blue) and bridging linkers (green) both decay toward zero as the valency κ increases, while the number of unbound ligands $\bar{n}_l$ (light green) converges to a finite value. This confirms that the effective interaction becomes dominated by the unbound-site entropy at large κ [cf. Eq. (S55)

]

E. Large linker valency limit

The large-valency limit is the most physically important regime for the main text results, as it reveals the exponential dependence of the adsorption threshold on valency at fixed total binding strength $\kappa f_{l/r} = \text{const}$. In this limit, $\kappa_\gamma \to \infty$, and each linker can form many bonds per arm. We show below that: (i) the numbers of adsorbed and bridging linkers vanish, so that the effective interaction is controlled entirely by the unbound-site entropy; and (ii) the adsorption threshold decreases exponentially with κ.

1. Vanishing of linker populations

At large valency, the average number of bonds that a single linker forms with species γ grows linearly with κ_γ:

$$\kappa_{{\rm link},\gamma} = \frac{\kappa_\gamma \bar{n}_\gamma \xi_\gamma (\bar{n}_\gamma \xi_\gamma + 1)^{\kappa_\gamma - 1}}{(\bar{n}_\gamma \xi_\gamma + 1)^{\kappa_\gamma} - 1} \approx \frac{\kappa_\gamma \bar{n}_\gamma \xi_\gamma}{\bar{n}_\gamma \xi_\gamma + 1} \,. \tag{S53}$$

The key physical consequence is that, while each linker binds to an increasing number of sites, the total number of sites does not change. The number of distinct linker molecules must therefore decrease. Specifically, the total numbers of dangling and bridging linkers scale as

$$M_\gamma = \frac{\sum_i i\, m_\gamma^{(i)}}{\kappa_{{\rm link},\gamma}} \to 0 \,, \qquad Q = \frac{\sum_{i,j} i\, b^{(ij)}}{\kappa_{{\rm link},\gamma}} \to 0 \,, \tag{S54}$$

because the numerators remain finite while the denominators diverge. Fig. S2 illustrates this behavior numerically: as κ increases, both the numbers of adsorbed and bridging linkers decay to zero, while the unbound number $\bar{n}_\gamma$ converges to a finite value.

2. Entropy-dominated effective interaction

Since the linker populations vanish, the dangling and bridging contributions to $F_{\rm eff}$ become negligible, and the effective interaction reduces to the unbound-site entropy alone:

$$\beta F_{\rm eff} \simeq \sum_{\gamma \in \{l,r\}} \left[n_\gamma \log \frac{\bar{n}_\gamma}{n_\gamma} - (\bar{n}_\gamma - n_\gamma) \right]. \tag{S55}$$

Crucially, this expression contains no explicit dependence on the valency κ. A given value of θ therefore corresponds to a specific pair $(\bar{n}_l, \bar{n}_r)$ that is independent of κ as θ is a function of $\beta F_{\rm eff}$. The entire valency dependence of the adsorption threshold enters through the self-consistent equations, which determine at what linker chemical potential μ the required $(\bar{n}_l, \bar{n}_r)$ pair is realized.

3. Adsorption threshold at large valency

At the adsorption threshold, linkers are sparse and bridging dominates over dangling (cf. Sec. III B). In the large-κ limit, the self-consistent equations require

$$\bar{n}_\gamma + e^{\beta\mu-\beta f_{\rm cnf}}\,\kappa_\gamma \bar{n}_\gamma \xi_\gamma\,(\bar{n}_\gamma\xi_\gamma+1)^{\kappa_\gamma-1}\,(\bar{n}_\omega\xi_\omega+1)^{\kappa_\omega} = n_\gamma\,, \tag{S56}$$

where the dangling terms have been dropped since bridging is exponentially favored when both $(\bar{n}_l\xi_l+1)^{\kappa_l}$ and $(\bar{n}_r\xi_r+1)^{\kappa_r}$ are large [cf. Eq. (S9) vs. Eq. (S8)]. Solving for the adsorption chemical potential yields

$$\beta\mu_{\rm ad} \approx \log\frac{(n_\gamma-\bar{n}_\gamma)(\bar{n}_\gamma+1)}{\kappa_\gamma\bar{n}_\gamma\, e^{-\beta f_l\frac{\bar{n}_l}{1+\bar{n}_l}-\beta f_r\frac{\bar{n}_r}{1+\bar{n}_r}}\,(\bar{n}_l+1)^{\kappa_l}(\bar{n}_r+1)^{\kappa_r}}\,, \tag{S57}$$

where the pair $(\bar{n}_l,\bar{n}_r)$ satisfying $\theta=\theta_{\rm th}$ is determined from Eq. (S55) together with the constraint

$$\frac{\kappa_l\bar{n}_l\xi_l(\bar{n}_r\xi_r+1)(n_r-\bar{n}_r)}{\kappa_r\bar{n}_r\xi_r(\bar{n}_l\xi_l+1)(n_l-\bar{n}_l)} = 1\,, \tag{S58}$$

obtained by requiring equal chemical potentials from the two self-consistent equations.

The crucial observation is that the $(\bar{n}_l+1)^{\kappa_l}(\bar{n}_r+1)^{\kappa_r}$ factor in the denominator of Eq. (S57) grows exponentially with κ_l and κ_r, while all other factors are independent of valency or less dominant. This gives

$$\frac{\Delta\beta\mu_{\rm ad}}{\Delta\kappa} = -\big[\log(\bar{n}_l+1)+\log(\bar{n}_r+1)\big]\,, \tag{S59}$$

if we assume $\kappa_l=\kappa_r=\kappa$. This establishes the exponential decrease of the adsorption threshold with valency stated in Eq. (5) of the main text.

4. Desorption threshold at large valency

On the desorption side at large valency, binding sites are nearly fully saturated. The unbound numbers scale inversely with valency, $\bar{n}_\gamma \sim L_\gamma/(\xi_\gamma\kappa_\gamma)$, where L_γ is a finite constant. Substituting this scaling ansatz into the self-consistent equations, the adsorbed-state equations become

$$e^{\beta\mu_{\rm dp}}\,L_l\,e^{L_l}\left[1+e^{-\beta f_{\rm cnf}}(e^{L_r}-1)\right] = n_l\,, \tag{S60}$$

$$e^{\beta\mu_{\rm dp}}\,L_r\,e^{L_r}\left[1+e^{-\beta f_{\rm cnf}}(e^{L_l}-1)\right] = n_r\,, \tag{S61}$$

and the desorbed-state equations simplify to

$$e^{\beta\mu_{\rm dp}}\,L_l'\,e^{L_l'} = n_l\,,\qquad e^{\beta\mu_{\rm dp}}\,L_r'\,e^{L_r'} = n_r\,. \tag{S62}$$

The desorption threshold $\mu_{\rm dp}$ is determined together with L_l, L_r, L_l', L_r' by solving these four equations jointly with the condition

$$\beta F_{\rm eff} = -\log\left[1+\frac{\theta_{\rm th}}{(1-\theta_{\rm th})z_g}\right] \approx \sum_{\gamma\in\{l,r\}} n_\gamma \log\frac{L_\gamma}{L_\gamma'}\,. \tag{S63}$$

These equations are solved numerically; the key physical result is that $\mu_{\rm dp}$ converges to a plateau at large κ (cf. Fig. 2d of the main text). This saturation occurs because, once all binding sites are fully occupied, further increasing the number of binding arms per linker does not create additional bridges and therefore has no effect on the desorption behavior. When the Poisson distribution of n_r is included, the desorption threshold is obtained by requiring $\langle\theta\rangle=\theta_{\rm th}$ following the same procedure, using the inter-site relations Eqs. (S64) and (S65) discussed in Sec. IV.

IV. ADSORPTION AND DESORPTION THRESHOLDS WITH POISSON-DISTRIBUTED RECEPTORS

In the preceding sections, the receptor number n_r at each site was treated as fixed. In practice, however, receptors are randomly distributed on the substrate, and different sites carry different numbers of receptors. In this section, we account for this heterogeneity by treating n_r as a Poisson-distributed random variable with mean $\langle n_r\rangle$, and show how the adsorption and desorption thresholds are determined in this ensemble setting.

TABLE I: Summary of leading-order scaling behaviors in each limiting regime. Here $\rho = e^{\beta\mu}$ is the linker density and $\xi_\gamma = e^{-\beta f_\gamma}$ the single-bond partition function. The symmetric protocol $\kappa_l = \kappa_r = \kappa$ is assumed where relevant.

Regime	scaling	α_ρ	Reference
Weak binding ($\xi_{l/r} \to 0$)	$\theta \sim \xi_l \, \xi_r$	—	Eq. (S35)
Low density ($\rho \to 0$)	$\theta \sim \rho$	1	Eq. (S39)
High density ($\rho \to \infty$)	$\theta \sim \rho^{-1}$	−1	Eq. (S42)
Maximal bridging, adsorption side	$\theta \sim \rho^{\min(n_l, n_r)/\kappa}$	$\min(n_l, n_r)/\kappa$	Eq. (S46)
Maximal bridging, desorption side	$\theta \sim \rho^{-\min(n_l, n_r)/\kappa}$	$-\min(n_l, n_r)/\kappa$	Eq. (S52)
Large valency ($\kappa \to \infty$)	$\log \rho_{\rm ad} \sim -[\log(\bar{n}_l{+}1) + \log(\bar{n}_r{+}1)]\,\kappa$	—	Eq. (S59)

A. Inter-site relations between self-consistent solutions

A key simplification arises from the fact that all sites share the same linker chemical potential μ. This means that the self-consistent solutions $(\bar{n}_l, \bar{n}_r)$ for a site with receptor number n_r are not independent of the solutions $(\bar{n}_l^*, \bar{n}_r^*)$ for a different site with receptor number n_r^*; instead, equating the chemical potentials from the self-consistent equations for both sites and rearranging yields the exact relations

$$\frac{\frac{n_l - \bar{n}_l}{\bar{n}_l}\,(\bar{n}_l\xi_l + 1)}{\frac{n_l^* - \bar{n}_l^*}{\bar{n}_l^*}\,(\bar{n}_l^*\xi_l + 1)} = \frac{(\bar{n}_l\xi_l + 1)^{\kappa_l}\,(\bar{n}_r\xi_r + 1)^{\kappa_r}}{(\bar{n}_l^*\xi_l + 1)^{\kappa_l}\,(\bar{n}_r^*\xi_r + 1)^{\kappa_r}}\,, \tag{S64}$$

$$\frac{\frac{n_r - \bar{n}_r}{\bar{n}_r}\,(\bar{n}_r\xi_r + 1)}{\frac{n_r^* - \bar{n}_r^*}{\bar{n}_r^*}\,(\bar{n}_r^*\xi_r + 1)} = \frac{(\bar{n}_l\xi_l + 1)^{\kappa_l}\,(\bar{n}_r\xi_r + 1)^{\kappa_r}}{(\bar{n}_l^*\xi_l + 1)^{\kappa_l}\,(\bar{n}_r^*\xi_r + 1)^{\kappa_r}}\,. \tag{S65}$$

These relations are exact at any valency and express a physically intuitive fact: because sites are coupled through the shared bulk linker reservoir, the equilibrium binding state at one site relates to the binding state at every other site. In practice, this means that once the self-consistent equations are solved for a single reference pair (n_l, n_r), the solutions for all other receptor numbers n_r^* can be obtained from Eqs. (S64)–(S65) without resolving the full self-consistent equations again.

B. Simplification in the large-valency limit

In the large-valency limit ($\kappa_l, \kappa_r \to \infty$), the inter-site relations simplify considerably. Since both $(\bar{n}_\gamma\xi_\gamma + 1)^{\kappa_\gamma}$ and the right-hand side of Eqs. (S64)–(S65) must converge to finite values, the κ_l-th root of the right-hand side approaches unity, giving

$$\frac{(\bar{n}_l\xi_l + 1)\,(\bar{n}_r\xi_r + 1)^{\kappa_r/\kappa_l}}{(\bar{n}_l^*\xi_l + 1)\,(\bar{n}_r^*\xi_r + 1)^{\kappa_r/\kappa_l}} = \sqrt[\kappa_l]{\frac{\frac{n_r - \bar{n}_r}{\bar{n}_r}\,(\bar{n}_r\xi_r + 1)}{\frac{n_r^* - \bar{n}_r^*}{\bar{n}_r^*}\,(\bar{n}_r^*\xi_r + 1)}} \approx 1\,, \tag{S66}$$

and

$$\frac{\frac{n_l - \bar{n}_l}{\bar{n}_l}\,(\bar{n}_l\xi_l + 1)}{\frac{n_l^* - \bar{n}_l^*}{\bar{n}_l^*}\,(\bar{n}_l^*\xi_l + 1)} = \frac{\frac{n_r - \bar{n}_r}{\bar{n}_r}\,(\bar{n}_r\xi_r + 1)}{\frac{n_r^* - \bar{n}_r^*}{\bar{n}_r^*}\,(\bar{n}_r^*\xi_r + 1)}\,. \tag{S67}$$

Under the symmetric protocol $\kappa_l = \kappa_r = \kappa$, Eq. (S66) has a direct and important consequence: the quantity $\log(\bar{n}_l + 1) + \log(\bar{n}_r + 1)$ takes the same value for all sites, regardless of the specific receptor number n_r—provided that $\bar{n}_\gamma$ remains finite and bounded away from n_γ (i.e., the site is neither completely unbound nor completely saturated). This invariance is what ensures a universal slope $\Delta\beta\mu_{\rm ad}/\Delta\kappa$ across all sites in the ensemble.

C. Determining the thresholds in the Poisson ensemble

With the inter-site relations in hand, the adsorption and desorption thresholds are determined as follows.

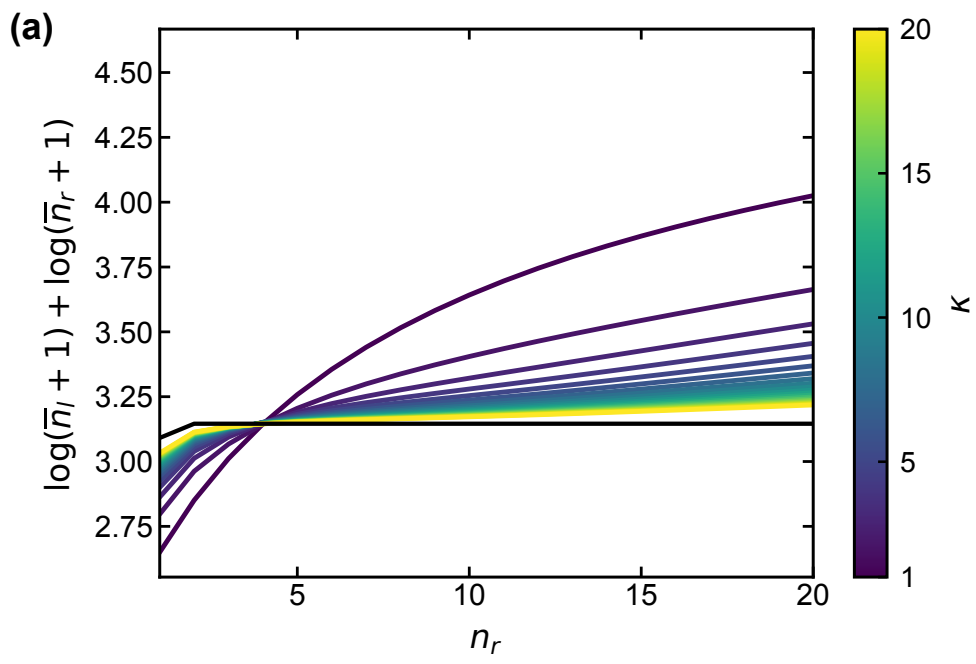

FIG. S3: **Convergence to Eq. S71 over large valency**. The quantity $\log(\bar{n}_l + 1) + \log(\bar{n}_r + 1)$, which determines the slope $\Delta\beta\mu_{\rm ad}/\Delta\kappa$ [Eq. (S70)], is plotted as a function of receptor number n_r for several finite valencies κ (colored curves) and compared with the large-κ asymptote Eq. (S71) (black curve). With increasing κ, the finite-valency curves converge toward the asymptotic relation. Notably, the curves for different κ nearly intersect at a common point, suggesting that the slope $\Delta\beta\mu_{\rm ad}/\Delta\kappa$ is approximately κ-independent even at moderate valency.

a. Adsorption threshold. The ensemble-averaged adsorption probability is

$$\theta = \big\langle \theta(n_r) \big\rangle_{\langle n_r \rangle} = \sum_{n_r=0}^{\infty} P(n_r; \langle n_r \rangle)\, \theta(n_r)\,, \tag{S68}$$

where $P(n_r; \langle n_r \rangle)$ is the Poisson probability mass function and $\theta(n_r)$ is the adsorption probability at a site with n_r receptors, computed from the Langmuir isotherm Eq. (3) of the main text. The adsorption threshold $\mu_{\rm ad}$ is defined by the condition $\theta = \theta_{\rm th}$.

In the large-valency limit, the adsorption threshold follows

$$\beta\mu_{\rm ad} \approx \log \frac{(n_l - \bar{n}_l)(\bar{n}_l + 1)}{\kappa_l \bar{n}_l\, e^{-\beta f_l \frac{\bar{n}_l}{1+\bar{n}_l} - \beta f_r \frac{\bar{n}_r}{1+\bar{n}_r}}\, (\bar{n}_l + 1)^{\kappa_l} (\bar{n}_r + 1)^{\kappa_r}}\,, \tag{S69}$$

where $(\bar{n}_l, \bar{n}_r)$ is any pair from the set of solutions satisfying $\theta = \theta_{\rm th}$ in the Poisson ensemble. The freedom to choose any such pair follows from the inter-site invariance established in Sec. IV B: since the chemical potential is uniform across sites, $\Delta\beta\mu_{\rm ad}/\Delta\kappa$ takes the same value for every site with finite $\bar{n}_\gamma$ and $n_\gamma - \bar{n}_\gamma$. In practice, the set of solutions corresponding to $\theta = \theta_{\rm th}$ is found numerically using the bisection method over μ.

As discussed in Sec. III E 2, the effective interaction Eq. (S55) has no explicit κ dependence, so that $\theta = \theta_{\rm th}$ corresponds to a specific set $\{(\bar{n}_l, \bar{n}_r)\}$ that is independent of valency. The slope of the adsorption threshold with respect to valency is therefore the constant

$$\frac{\Delta\beta\mu_{\rm ad}}{\Delta\kappa} = -\big[\log(\bar{n}_l + 1) + \log(\bar{n}_r + 1)\big]\,, \tag{S70}$$

which, by virtue of Eq. (S66), is the same for every site in the ensemble. The convergence of $\log(\bar{n}_l + 1) + \log(\bar{n}_r + 1)$ to a well-defined value in the large-κ limit is illustrated in Fig. S3. Specifically, the limiting value satisfies

$$\lim_{\kappa\to\infty} \big[\log(\bar{n}_l + 1) + \log(\bar{n}_r + 1)\big] = \min\big[\log(n_l + 1) + \log(n_r + 1),\; C\big]\,, \tag{S71}$$

where C is a constant determined by the parameters of the system. As Fig. S3 shows, the curves for different finite valencies appear to pass through a common point, suggesting that $\Delta\mu/\Delta\kappa$ remains approximately constant even at moderate κ values.

We note that the linear relationship between $\beta\mu_{\rm ad}$ and κ is strictly valid only in the large-κ limit. The unexpectedly good linearity observed at moderate κ (cf. Fig. 2d of the main text and Fig. S4) can be understood from the structure of the effective interaction: the unbound-site entropy terms $\sum_\gamma [n_\gamma \log(\bar{n}_\gamma/n_\gamma) + n_\gamma - \bar{n}_\gamma]$, which have no explicit κ dependence, dominate the effective interaction even at moderate valency, while the dangling and bridging linker contributions remain subdominant.

b. Adsorption threshold at low saturation When the detection threshold $\theta_{\rm th}$ is very low, the effective interaction at the adsorption point is correspondingly small, and the adsorption probability simplifies to $\theta \approx z_g\, \beta F_{\rm eff}$ [cf. Eq. (S39)]. Averaging over the Poisson-distributed receptors gives

$$\theta = \langle z_g\, \beta F_{\rm eff} \rangle\,, \tag{S72}$$

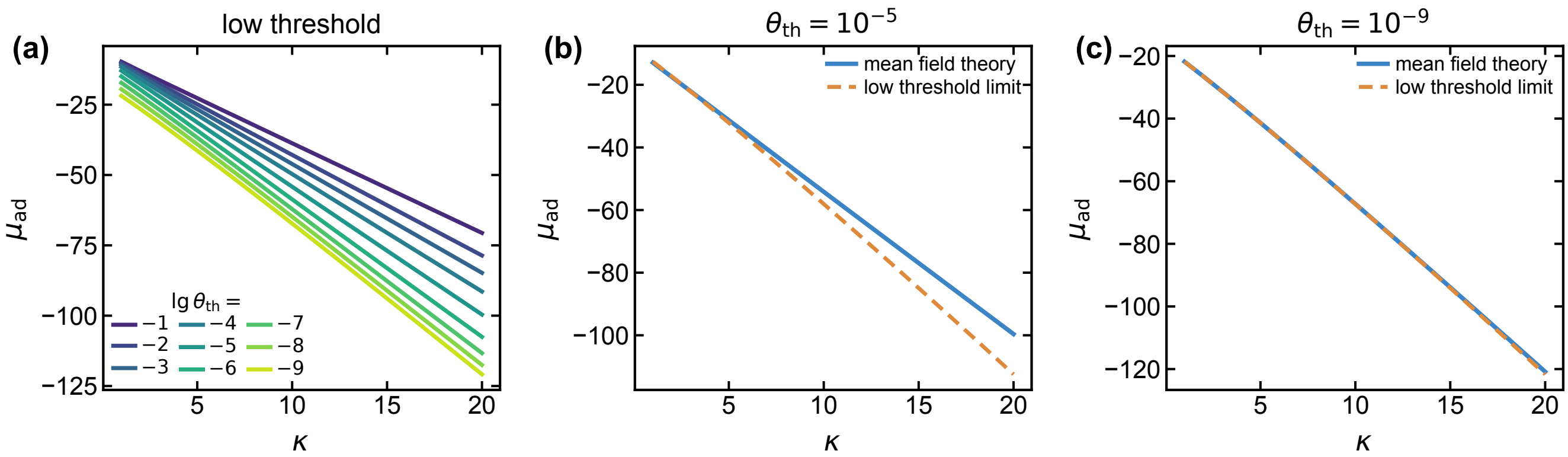

FIG. S4: **Adsorption threshold versus valency at low detection thresholds.** Parameters: $\kappa\beta f_l = \kappa\beta f_r = -5$, $\beta f_{\rm cnf} = 2$, $n_l = \langle n_r \rangle = 10$. (a) Adsorption chemical potential $\mu_{\rm ad}$ versus valency κ for detection thresholds ranging from $\theta_{\rm th} = 10^{-1}$ to 10^{-9}. (b), (c) Comparison of the full mean-field theory (solid curves) with the low-threshold approximation Eq. (S73) (dashed curves) for $\theta_{\rm th} = 10^{-5}$ (b) and 10^{-9} (c). The approximation becomes increasingly accurate at lower thresholds, where the effective interaction at the adsorption point is weak and $\theta \approx z_g\,\beta F_{\rm eff}$ holds.

from which the adsorption chemical potential follows as

$$\beta\mu_{\rm ad} \approx \log \frac{\theta_{\rm th}}{z_g\, e^{-\beta f_{\rm cnf}}\,(n_l\xi_l + 1)^{\kappa_l}\,\langle (n_r\xi_r + 1)^{\kappa_r}\rangle}\,. \tag{S73}$$

This expression shows that the adsorption threshold shifts nonlinearly with κ at small valency if $\kappa = \kappa_l = \kappa_r$, but $\Delta\beta\mu_{\rm ad}/\Delta\kappa$ converges to the constant given by the large-κ analysis as κ increases (Fig. S4). The low-threshold formula Eq. (S73) agrees well with the full mean-field theory when $\theta_{\rm th}$ is sufficiently small (Fig.S4).

c. Desorption threshold. The desorption threshold $\mu_{\rm dp}$ is determined by the same procedure: find the chemical potential at which $\langle\theta\rangle = \theta_{\rm th}$ on the desorption side of the adsorption curve. The inter-site relations Eqs. (S64)–(S65) (or their large-κ simplifications) are used to propagate the solution from one reference n_r to all others, and the threshold is located numerically via bisection. As discussed in Sec. III E 4, $\mu_{\rm dp}$ converges to a plateau at large κ because full saturation of binding sites renders additional binding arms per linker ineffective.

[S1] X. Xia, Y. Peng, K. Li, and R. Ni, Rep. Prog. Phys. **88**, 078101 (2025).
[S2] X. Xia and R. Ni, Phys. Rev. Lett. **132**, 118202 (2024).
